# Naturally occurring circadian rhythm variation associated with clock gene loci in Swedish *Arabidopsis* accessions

**DOI:** 10.1111/pce.13941

**Published:** 2021-01-11

**Authors:** Hannah Rees, Ryan Joynson, James K. M. Brown, Anthony Hall

**Affiliations:** ^1^ Organisms and Ecosystems Earlham Institute, Norwich Research Park Norwich UK; ^2^ Institute of Integrative Biology, University of Liverpool Liverpool UK; ^3^ Crop Genetics John Innes Centre, Norwich Research Park Norwich UK

**Keywords:** 1001 genomes project, Circadian clock, *COR28*, *ELF3*, GWA‐mapping, natural variation, Sweden, temperature compensation

## Abstract

Circadian clocks have evolved to resonate with external day and night cycles. However, these entrainment signals are not consistent everywhere and vary with latitude, climate and seasonality. This leads to divergent selection for clocks which are locally adapted. To investigate the genetic basis for this circadian variation, we used a delayed fluorescence imaging assay to screen 191 naturally occurring Swedish *Arabidopsis* accessions for their circadian phenotypes. We demonstrate that the period length co‐varies with both geography and population sub‐structure. Several candidate loci linked to period, phase and relative amplitude error (RAE) were revealed by genome‐wide association mapping and candidate genes were investigated using TDNA mutants. We show that natural variation in a single non‐synonymous substitution within *COR28* is associated with a long‐period and late‐flowering phenotype similar to that seen in TDNA knock‐out mutants. *COR28* is a known coordinator of flowering time, freezing tolerance and the circadian clock; all of which may form selective pressure gradients across Sweden. We demonstrate the effect of the *COR28*‐58S SNP in increasing period length through a co‐segregation analysis. Finally, we show that period phenotypic tails remain diverged under lower temperatures and follow a distinctive “arrow‐shaped” trend indicative of selection for a cold‐biased temperature compensation response.

## Abstract 2

Circadian rhythms in Swedish *Arabidopsis* accessions vary with both geographical regions and population structure. Through GWA mapping, we find naturally occurring period length variation is associated with a single SNP within *COR28*. Finally, we show that phenotypic tails for period remain diverged at reduced temperatures and follow a distinctive arrow‐shaped trend.

## 1 INTRODUCTION

Plants are highly adapted to survive and exploit the daily fluctuations in light and temperature experienced as the earth spins on its axis. The circadian clock plays an intrinsic role in this; integrating temporal cues from the environment to inform photosynthetic, metabolic and developmental processes (Covington, Maloof, Straume, Kay, & Harmer, 2008; Harmer et al., 2000). A robustly oscillating circadian clock which is highly synchronized to external day‐length contributes to the overall fitness of the plant, giving it an edge over competitors, predators and pathogens (Dodd et al., 2005; Green, Tingay, Wang, & Tobin, 2002; Ingle et al., 2015; Michael et al., 2003). Circadian rhythms can be quantified by their period (the time taken to complete one cycle), their phase (the time of day in which the rhythm peaks), their amplitude (the change in intensity from a baseline) and their relative amplitude error (RAE) which is the amplitude error divided by the overall amplitude of the rhythm and can be equated to rhythm robustness. In our study, circadian phenotyping was achieved using delayed fluorescence (DF) imaging. Delayed fluorescence levels are circadian regulated and reflect the changes in the photosynthetic state of photosystem II (Gould et al., 2009). DF imaging has been validated as a reliable and flexible tool to measure circadian rhythms in a range of plant models but has not previously been used for phenotyping of a large population of individuals on the scale required for genome‐wide association mapping.

*Arabidopsis thaliana* is a model plant system which has been extensively studied in circadian biology. The *Arabidopsis* clock is comprised of a series of interlocking negative transcriptional feedback loops connected by key activators that control the oscillation of clock gene expression (Hernando, Romanowski, & Yanovsky, 2017). Key genes within this network include *CIRCADIAN CLOCK ASSOCIATED 1 (CCA1)* and *LATE ELONGATED HYPOCOTYL (LHY)* which transcriptionally repress *TIMING OF CAB EXPRESSION (TOC1)* (Alabadi et al., 2001). Downstream genes interact with the clock to communicate circadian rhythmicity to physiological outputs. Examples include photoperiodic regulation of flowering time and the temporal gating of cold acclimation responses (Kinmonth‐Schultz, Golembeski, & Imaizumi, 2013). Flowering under long days is instigated through accumulation of the floral promoter CONSTANS (CO) controlled by the circadian clock component GIGANTEA (Suárez‐López et al., 2001). In winter‐annuals, flowering is also dependent on the vernalization response of *FLOWERING LOCUS C (FLC)* and *FRIGIDA (FRI)* (Bastow et al., 2004; Johanson et al., 2000). Cold tolerance is diurnally activated through alternative splicing of *CCA1* which co‐regulates the expression of *COLD REGULATED (COR)* genes alongside light and temperature stress pathways (James et al., 2012; Kinmonth‐Schultz et al., 2013).

The endogenous core circadian network is entrained by external stimuli; most notably light and temperature. Day‐length, light composition and light intensity have all been shown to affect circadian rhythms (Aschoff, 1979; Más, Alabadí, Yanovsky, Oyama, & Kay, 2003; Pittendrigh & Minis, 1964; Yanovsky & Kay, 2002). Temperature also has a well‐documented effect on entrainment of these rhythms, (Edwards, Lynn, Gyula, Nagy, & Millar, 2005; Gould et al., 2006; Salome & McClung, 2005) with a degree of period shortening expected under higher temperatures. Circadian rhythms are said to be “temperature compensated”; they resist large changes in period‐length in response to temperature (Pittendrigh, 1954). The extent of temperature compensation has been shown to vary between *Arabidopsis* accessions (Gould et al., 2006; Kusakina, Gould, & Hall, 2014). Rhythm robustness is also strongly affected by temperature, although the temperature which produces the most rhythmic oscillations appears to be dependent on the species and circadian assay used (Kusakina et al., 2014; Rees et al., 2019). Plants with clocks which resonate with environmental conditions are typically fittest, however it has been suggested that in climates with large seasonality there may be a compromise for clocks which are adaptable to rapidly changing day‐lengths (Michael et al., 2003).

Natural populations of several important model organisms that exhibit extensive diversity in their circadian behaviour have been documented. Pupal eclosion rhythms of Drosophila are latitude dependent; with shorter rhythms, earlier phases and less robust rhythms observed in Northern latitudes (Allemand & David, 1976; Lankinen, 1986). In *Arabidopsis*, Michel et al. conducted a global study of leaf movement rhythms in 150 accessions and found that day‐length of origin country correlated positively with period, but not phase or amplitude. They also identified several loci in the *TOC1/PRR* family which determined natural variation in period, phase and amplitude independently (Michael et al., 2003). Other investigations have shown allelic diversity of several clock related genes including *FLC* (Swarup et al., 1999) *GI* (de Montaigu et al., 2015) and *EARLY FLOWERING 3 (ELF3)* (Anwer et al., 2014) which contribute to natural circadian phenotypes without fully disrupting the clock mechanism. The positive relationship with period and latitude has also been observed in natural populations of *Mimulus guttatus* (Greenham et al., 2017) and in cultivated varieties of soybean and tomato (Greenham et al., 2017; Müller et al., 2016), potentially due to unintentional selection for circadian rhythms which function best at particular latitudes.

In this study, we focused on a collection of *Arabidopsis* accessions from across a large latitudinal range in Sweden; a country with variations in climatic, anthropogenic and day‐length factors all possibly influencing clock adaption. Northern latitudes around 63°N have permanent snow cover during winter months and the growing season is much cooler and shorter than in the South. At the solstice, there is almost 3 hr difference in day‐length between the North and South. This divergence of climate has led to selection for ecotypes with adapted growth and flowering strategies (Bloomer & Dean, 2017; Shindo et al., 2005). Analysis of the global population structure of *Arabidopsis* accessions has previously identified Swedish accessions as being genetically distinct from the wider population, with further differentiation within the country between North and South (Horton et al., 2012; Huber, Nordborg, Hermisson, & Hellmann, 2014; Long et al., 2013; Nordborg et al., 2005). Accessions from South Sweden have high genetic diversity within a relatively small area, perhaps suggesting a historic emigration from central Europe following glacial retreat (Alonso‐Blanco et al., 2016). Northern Swedish accessions have lower genetic diversity but larger genome sizes (Long et al., 2013) and also carry a surprising reservoir of drought tolerance genes (Exposito‐Alonso et al., 2018). Completion of the 1001 genomes project in 2006 has facilitated a recent expansion in *Arabidopsis* genome wide association (GWA) studies made possible through the provision of high‐quality re‐sequenced genotype data. These accessions are publicly available, geo‐referenced and genetically inbred making it easy for researchers to perform experiments over several generations under a variety of conditions (Weigel & Mott, 2009). Many of the accessions in this study have also been characterized for other phenotypic traits such as seed dormancy (Kerdaffrec et al., 2016), flowering time (Sasaki, Zhang, Atwell, Meng, & Nordborg, 2015), and freezing tolerance (Horton, Willems, Sasaki, Koornneef, & Nordborg, 2016).

The objectives for this research were firstly: to investigate whether circadian diversity exists in Swedish *Arabidopsis* and to understand to what extent this variation can be explained by either latitude or the underlying population structure. We next wanted to identify and validate genetic variation in candidate genes which might explain the observed circadian variation. To our knowledge, this work provides the first example of a GWA study using circadian phenotypes in plants. Our final objective was to measure circadian rhythms under 10°C and 16°C to test whether divergence of circadian traits persists across a range of temperatures.

## 2 METHODS

### 2.1 Plant material, plate set‐up and growth conditions

Seed for the 192 *Arabidopsis* accessions were gas sterilized using 3 ml hydrochloric acid in 100 ml sodium hypochlorite. We then stratified seed in 1 ml of sterile water for 2 days at 4°C before plating. Clear 96‐well plates with flat bottomed wells (Thermo Scientific, cat no: 10287631) were filled with 250 μl of Murashige and Skoog agar media (1.5% Agar, pH 5.8, no sucrose). Approximately 20 seeds were added to each well of the plates following a randomized Alpha‐lattice design (see Extended Methods) and left to dry until all residual water had evaporated. Each accession was replicated 18 times across the 3 imaging runs. Clear microplate lids (Thermo Scientific, cat no: 10334311) were secured to each plate with micropore tape ensuring that the lid was raised slightly from the top of the wells to allow condensation to circulate freely. Plates were returned to the fridge for 2 more days before transfer to the growth cabinet set at 12:12 L:D cycle at 22°C under approximately 200 μmol m^−2^ s^−1^ white light for 14 days. Prior to the temperature response experiments, accessions from the 191 phenotyping screen were bulked for seed (see Extended Methods). Plates were all grown under 12:12 L:D at 22°C light for 10 days and were then transferred to the temperatures in which they would be imaged for 4 days.

Note: For the temperature and mutant screening experiments, the corner wells of the 96‐well plates were not used in the designs as we found that corners had significant effects on circadian rhythms in the 191 dataset; potentially due to these wells drying out more quickly (Figure S8).

### 2.2 Imaging conditions

Delayed fluorescence imaging was carried out using Lumo Reteiga CCD cameras (QImaging, Canada) housed in two temperature‐controlled cabinets. Six 96‐well plates can be imaged simultaneously in each cabinet. Imaging conditions were exactly those described for constant light (L:L) imaging of *Brassica* leaves in Rees et al., 2019. Images were captured every hour with a 1‐min exposure time.

### 2.3 FIJI ROI selection and circadian analysis

Image stacks were imported into FIJI (RRID:SCR_002285) (Schindelin et al., 2012) and regions of interest (ROI) were selected using a semi‐automated approach (full details in Extended Methods; macro and user guide in Supplementary Files 4 and 5). Measurements for integrated density were taken for these regions across the stack using the Multi‐measure plugin. The time‐series was converted to decimalized time relative to hours elapsed from entrained dawn (T0) and was cropped to 150 hr to standardize between experiments. Period estimation was done using the online platform Biodare2 (https://biodare2.ed.ac.uk) applying the FFT‐NLLS algorithm on a data window of 24–120 hr with expected periods set to 18–34 hr. Raw data was baseline and amplitude de‐trended prior to analysis. Manual inspection of resulting periods ensured that all arrhythmic traces were excluded from further analysis.

### 2.4 Data analysis and REML

We filtered period estimates from Biodare2 to exclude rhythms with RAE > 0.65 and period >28 hr. Imposing these cut‐offs drastically improved the reliability of period length predictions within each cultivar from an average *SD* of 9.66–1.28 hr, making an association analysis feasible. We next used restricted maximum likelihood (REML) to fit a linear mixed model to the 191 accession dataset and thus obtain accession means for period and RAE which were adjusted for the effects of cabinet and experimental run (see details in Extended Methods). An additional step was required to calculate phase means for each accession as the raw phase data is circular and relative to dawn (0) with a full circle representing 24 hr. Phase data was analysed with a circular regression model using the Genstat RCIRCULAR procedure to obtain accession mean phases adjusted for cabinet and run effects (Fisher & Lee, 1992).

For temperature experiments, no period or RAE cut‐offs were imposed as we predicted an increase in both these variables with lower temperatures (Dodd, Kusakina, Hall, Gould, & Hanaoka, 2014; Rees et al., 2019). We fitted linear models to the period and RAE data and identified the contribution of each component by analysis of variance (Tables S12–S14). The above data analysis was done using Genstat 18th edition (RRID:SCR_014595).

Map figures (Figures 2 and 4) were created using the ggmaps package in R using Google maps (2018) (Kahle & Wickham, 2013).

### 2.5 Principal component analysis

The PCA for genomic variation in the 191 Swedish accessions in Figure 2e was performed using a compressed mixed linear model run in R implementing the GAPIT package (Lipka et al., 2012; Zhang et al., 2010). Genotype data was downloaded from the 1001 genomes dataset filtered for bialleleic SNPs with >5% MAF (minor allele frequency). The first two PCs were used to split the accessions into the categories in Figure 2d,f.

### 2.6 Genome wide association study

Genome wide association mapping was conducted using an Accelerated Mixed Model (AMM) via the online web application GWA‐Portal (Seren, 2018). Accession means estimated from REML were used for period, phase and RAE with no further transformation necessary. Genotype data used was from the full‐sequence 1001 genomes dataset. Results were filtered for the top 10,000 −log_10_(*p*) values and then for hits with >5% MAF. Comparison to results from other models confirmed the major peaks and can be viewed in Figure S6.

### 2.7 Mutant screening

T‐DNA and EMS mutants were obtained from the Nottingham *Arabidopsis* Stock centre (RRID:SCR_004576) and primers designed using the iSect tool from the SIGnAL website. In addition, seed for *cor28‐2*, *cor27‐1* and *cor28‐2/cor27‐1* was kindly donated from Hongtao Liu's laboratory (Shanghai, China) where these mutants have previously been identified as long‐period using leaf‐movement and ProCCR2:LUC constructs (Li et al., 2016). *Cor28‐2* is a null mutant and *cor28‐1*, *cor27‐1* and *cor27‐2* are all knockdown mutants. Gene candidates, mutant IDs and primers used can be viewed in Supplementary File 2. All lines were genotyped to confirm homozygosity by PCR and gel electrophoresis prior to seed bulking other than *sco2* which was genotyped by Sanger sequencing to reveal the premature stop. We were unable to obtain or confirm homozygous mutants for *dnf* and *fab1c* and therefore these lines were not DF screened (Supplementary File 2). SALK lines for *elf3* were not obtained as the *elf3‐1* mutant has been previously confirmed in Box et al., 2015 as having a phenotype of reduced robustness using delayed fluorescence imaging (Box et al., 2015). WT control lines; Col‐0 and Ler were grown for seed in parallel to standardize seed quality. For *cor28‐1*, a homozygous WT control was derived by segregation of the original heterozygous TDNA line was used as a control. Five imaging experiments using a single camera and cabinet were carried out with each line present in at least three experiments. We did not apply a mixed model to the mutant screening data because only one camera was used.

### 2.8 Co‐segregation analysis

The co‐segregation analysis was conducted using a Cleaved Amplified Polymorphic Sequences (CAPS) assay to genotype and DF to phenotype crossed and selfed individual plants.

Primers were designed to amplify a 252bp fragment within *COR28* exon 2 (*COR28‐F*: 5′‐ACAGTAAGTAACCCCGAACC‐3′, *COR28‐R*: 5′‐TTCTTGACAAAAGTCCCACTC‐3′). These fragments were then digested using TaqI (Thermo Fisher Scientific, #ER0671, recognition site: T^CGA). TaqI was added to 10 μl of PCR reaction mixture and incubated at 65°C for 1.5 hr according to the manufacturer's instructions. Individuals homozygous for the *COR28* SNP 58S (found in the parent St‐0) are not recognized by TaqI and so presented as a single 252bp band on an agarose gel (3% Agarose, run for 1.5 hr at 50 V). Individuals homozygous for the *COR28* SNP 58W (found in the parent T800) were fully cleaved resulting in two bands 92bp and 160bp in length. Heterozygous individuals therefore had three bands at 252bp, 160bp and 92bp clearly separated by electrophoresis. Restriction digest gels for identifying F2 plants can be viewed in Supplementary File 6. All plants in generations (F_0_‐F_1_) were vernalized for 12 weeks at 4°C prior to growth at 22°C under long days (16:8 hr). Parent accessions St‐0 and T800 were genotyped to check for homozygosity prior to crossing. All three heterozygous F_1_ individuals were (♀T800 × ♂St‐0) and were also confirmed by the CAPS assay. These F_1_ plants were then selfed and the circadian rhythms of the F_2_ progeny were analysed using DF on detached leaves from 24‐day‐old plants grown under 12:12 hr light:dark conditions at 22°C. Leaves were placed on 0.5% agar plates and were exposed to 24 hr constant L:L prior to imaging as described for the 96‐well plates.

## 3 RESULTS

### 3.1 Quantitative circadian variation across 191 Swedish *Arabidopsis* accessions

**FIGURE 1:**
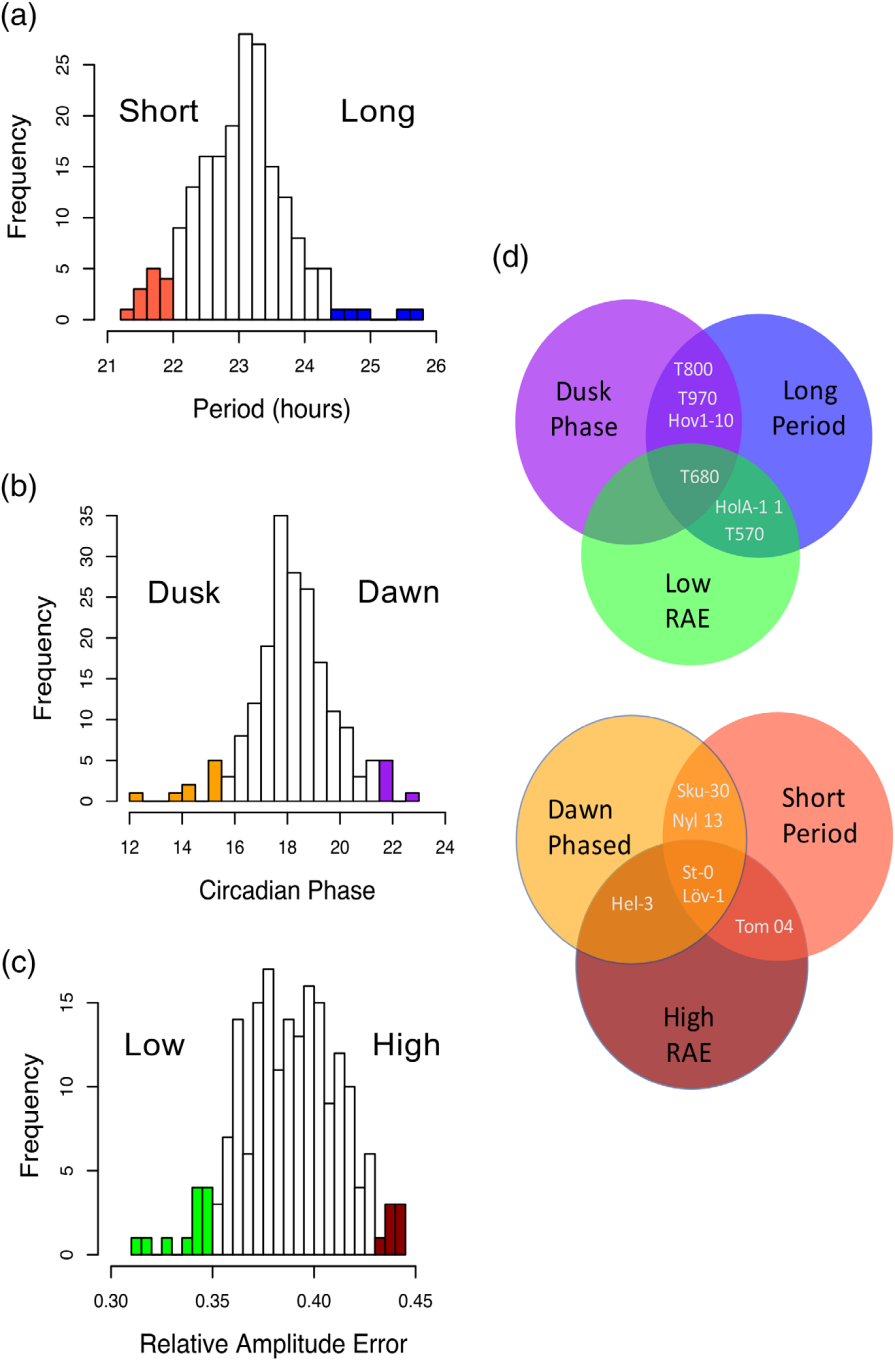
Circadian variation in 191 naturally occurring accessions from Sweden. Delayed fluorescence rhythms were characterized by period, phase and RAE and show significant variation (a–c). Colours represent the 10 most extreme accessions for each phenotype which we use as tail groups for further analysis. Some accessions belong to multiple tail groups as is shown in (d). This reflects the strong correlation between circadian characteristics (see Figure S4). Number of individual wells contributing to the mean of each accession ranged from 4 to 18, with each well representing the mean rhythms of approximately 15 seedlings [Colour figure can be viewed at wileyonlinelibrary.com]

We used delayed fluorescence imaging to characterize circadian rhythms in groups of 14‐day‐old seedlings entrained in 12 hr L 12 hr D cycles at 22°C and assayed under free‐running conditions of constant light (L:L) at 22°C. Accession means and *SE* adjusted for experimental effects were obtained using linear mixed models for period, amplitude and RAE and a circular regression model for phase (see Extended Methods). We found a 4.42 hr difference between the mean period of the fastest (21.28 hr, *SE* = 0.329) and slowest (25.70 hr, *SE* = 0.355) accessions tested (Figure 1a). The mean phase of peak DF intensity for each accession occurred over the dark half of the cycle (12–24/0 hr) with a huge difference (10.32 hr) from the earliest peaking (12.21 hr, *SE* = 0.58) to the latest (22.54 hr, *SE* = 0.20) (Figure 1b). This variation in period and phase is consistent with data previously reported from a global collection of *Arabidopsis* accessions (Michael et al., 2003). We also measured the robustness of these rhythms by looking at their RAE values. The most rhythmic (lowest RAE) mean was 0.31 (*SE* = 0.023) and the least rhythmic was 0.44 (*SE* = 0.024) representing approximately 20% of the possible range of this trait in this study (Figure 1c). Accession means for circadian phenotypes can be viewed in Supplementary File 1.

To assess the effect of genotype on each trait we performed a likelihood ratio test against a model which omitted genotype as a variance component. Including genotype in the mixed model had highly significant effects on period (*χ* ^2^ (1 d.f.) = 455.57, *p <* .0001) and RAE (*χ* ^2^ (1) = 65.97, *p <* .0001). Including genotype in the circular regression model also had a strongly significant effect on phase (*χ* ^2^ (191) = 407, *p <* .0001). Amplitude was analysed as a log_10_ transformation (see Extended Methods) and there was no significant effect of genotype on Log_10_Amplitude (*χ* ^2^ (1) = 0.19, *p =* 0.6). With this in mind, we dropped Amplitude as a trait of interest for the temperature and mutant screening experiments. REML output tables (Tables S1–S3), model checking graphs (Figures S1–S3) and log‐likelihood results (Tables S4–S7) are available to view.

We observed a strong correlation between the period, RAE and phase of each accession (Figure S4), especially between period and phase (adjusted *R* ^2^ = 0.43, *p <* .0001). We are unaware of any previously published correlation between natural variation in period and RAE. In this study, we found a highly significant negative relationship with longer periods having lower RAE scores (adjusted *R* ^2^ = 0.1, *p <* .0001).

We chose 10 accessions from the tails of the distributions of mean period, phase and RAE to create “phenotypic tail” groups representing the extremes of each trait. Some accessions represented two or more tail groups and could be split into two master groups of: long period, dusk phased, low RAE accessions and short period, dawn phased, high RAE accessions as explained in Figure 1d. These phenotypic tail groups were used for the temperature experiments described later in this paper (accessions in each tail are listed in Tables S8–S10).

Our next question was whether other quantified traits co‐varied with our circadian data. We used several previously published datasets to examine possible phenotypic relationships between our traits and flowering time (Li, Huang, Bergelson, Nordborg, & Borevitz, 2010; Sasaki et al., 2015), seed dormancy (Kerdaffrec et al., 2016) and freezing tolerance (Horton et al., 2016) (Table S11). Only flowering time data from Li et al. (2010) showed a moderate correlation with period (*p* < .01, *R* ^2^ = 0.28) and phase (*p* < .01, *R* ^2^ = 0.26). This flowering time data is based on flowering times recorded in conditions of variable day‐length reflecting annual conditions of a natural Swedish or Spanish climate (Li et al., 2010). However, no significant correlations were found between flowering times recorded under long‐days under either 10°C or 16°C by Horton et al. (2016), indicating that the relationship is highly dependent on the exact experimental conditions used.

**FIGURE 2:**
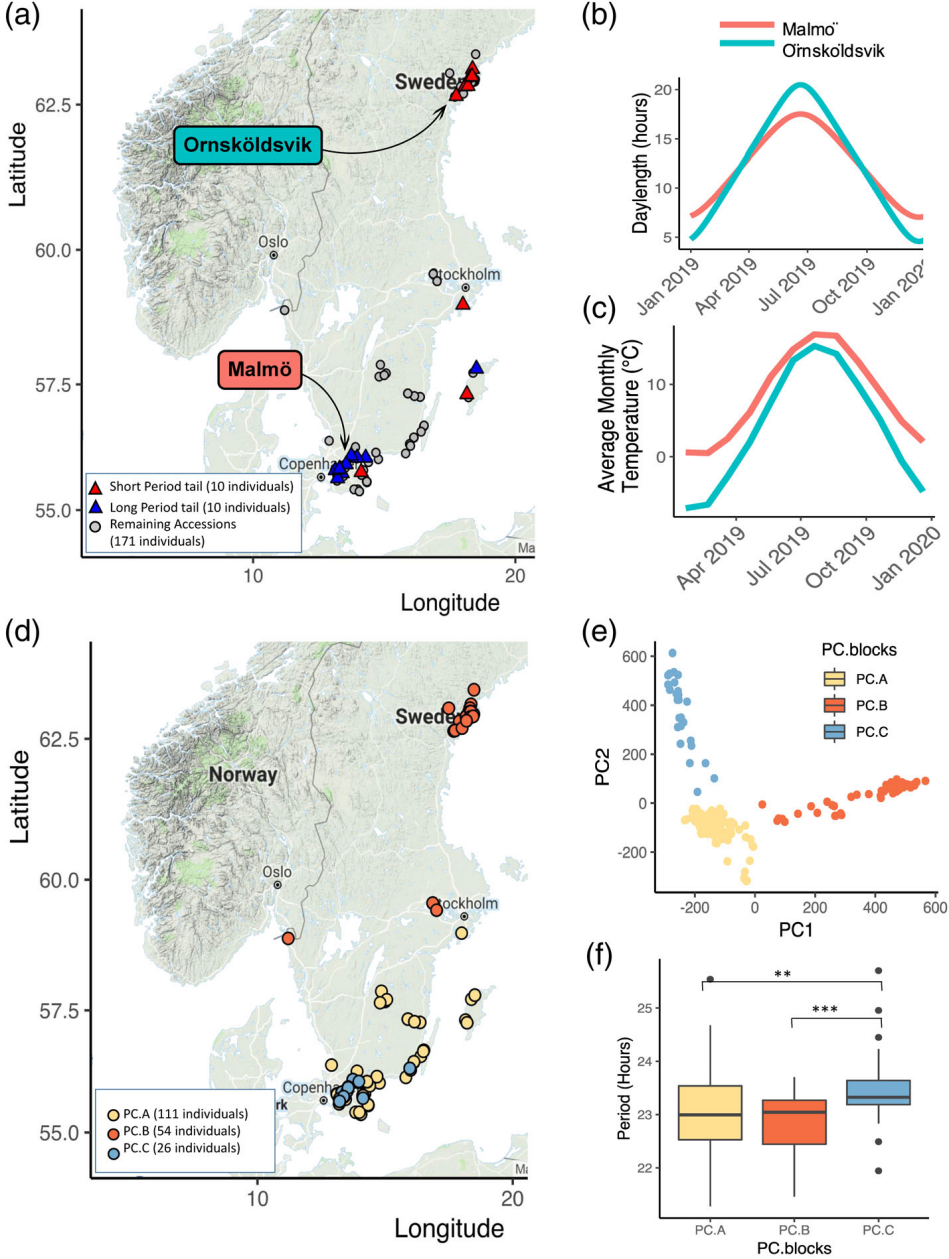
Period phenotypes are linked by both the geography and the genetic relatedness of accessions across Sweden. Figure (a) shows the location of origin of the 10 accessions with the shortest (red triangles) and longest (blue triangles) periods. The longest periods are found near the city of Malmo where day‐length and temperature fluctuates less throughout the year than in Ornskoldsvik where periods tend to be shorter (b, c). Day‐length and temperature averages were downloaded from timeanddate.com and are based on predictions for 2019 (Time and Date AS, 1995). PC analysis revealed a sub‐clade within the Southern Swedish accessions; PC.C coloured light blue in plots (d–f). This was used to distinguish accessions with significantly longer period phenotype than in the other PC groups; PC.A which represents the remaining southern accessions in yellow and PC.B which represents the northern accessions in red. Map figures were created using the ggmaps package in R using Google maps (accessed 2018) (Kahle & Wickham, 2013) [Colour figure can be viewed at wileyonlinelibrary.com]

We then investigated whether circadian traits varied according to the original location of the accessions. Period was found to be significantly correlated with longitude and latitude (*p <* .001), however a considerable amount of variation remained unexplained (adjusted *R* ^2^ < 0.1) (Figure S5). Despite this weak overall association with latitude, we observed an obvious segregation of accessions from the phenotypic tails of the period distribution, with the shortest period accessions tending to be located in Northern and mid‐latitude regions and the longest period accessions in the south (Figure 2a). No significant correlation with altitude was observed for these accessions.

Following on from this, we asked the question whether population sub‐structure could better explain the distribution of period phenotypes. We re‐classified the population into three groups split across the axes of the first two principal components (which together explain over 15% of the total genetic variation) (Figure 2d,e). PC.B broadly contained Northern accessions (*N* = 54, red points in Figure 2d). The Southern accessions split into two sub‐groups existing within the same geographic region; PC.A (*N* = 111) and PC.C (*N* = 26). We found that period is significantly longer in group PC.C (Figure 2f) (*F*(2,188) = 8.39, Tukey HSD for PC.A and PC.C *p <* .01, one‐way ANOVA). PC.A had mean periods similar to those obtained from Northern accessions (PC.B, Figure 2f). PC.C accessions also have significantly earlier phase peaks than those in PC.A (one‐way ANOVA, *F*(2,188) = 6.9149, Tukey HSD for PC.A and PC.C *p <* .001).

We removed the effects of PC groups from period length variation and found that the effect of latitude on the remaining period variation was much less significant (REML Model: Period ~ (1 | PC.blocks) + Latitude, applying Satterthwaite's method. *F*(1,7.23) = 8.87, *p* > .01). This indicates that period variation with latitude is due to the different locations of sub‐populations, rather than due to adaption following a latitudinal cline. Accessions in the long period tail group described in Figure 1a belonged to both PC.A (*N* = 6) and PC.C (*N* = 4), suggesting that long‐period traits may have been preserved in both sub‐populations in this area.

### 3.2 GWA mapping reveals loci associated with natural circadian variation

We used the online GWA‐portal to perform association mapping on the three circadian traits found to be significantly affected by genetic variation (Seren, 2018). We used an Accelerated Mixed Model (AMM) to account for population structure, although the other association models (linear and non‐parametric) gave similar results (see Figure S6). Pseudo‐heritability estimates for each trait were given as an output of the GWA analysis: period = 71%, RAE = 37% and phase = 13%.

**FIGURE 3:**
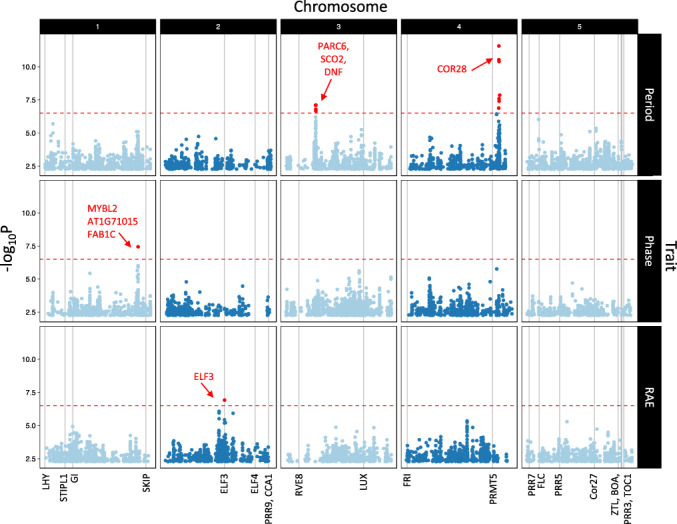
GWA analysis reveals candidate genes underlying natural variation in DF circadian phenotypes. Manhattan plots show AMM derived SNP associations with period (top panel), phase (middle panel) and RAE (bottom panel). Data shown has >5% MAF and reflects the 10,000 highest −log_10_(*p*) values. Gene candidates selected for further analysis are labelled in red with arrows. The red dotted line indicates the 6.5 −log_10_(*p*) arbitrary threshold used for this study. Vertical grey lines are labelled under the *x* axis and represent positions of known circadian and flowering time genes with altered circadian phenotypes previously reported in mutants [Colour figure can be viewed at wileyonlinelibrary.com]

**FIGURE 4:**
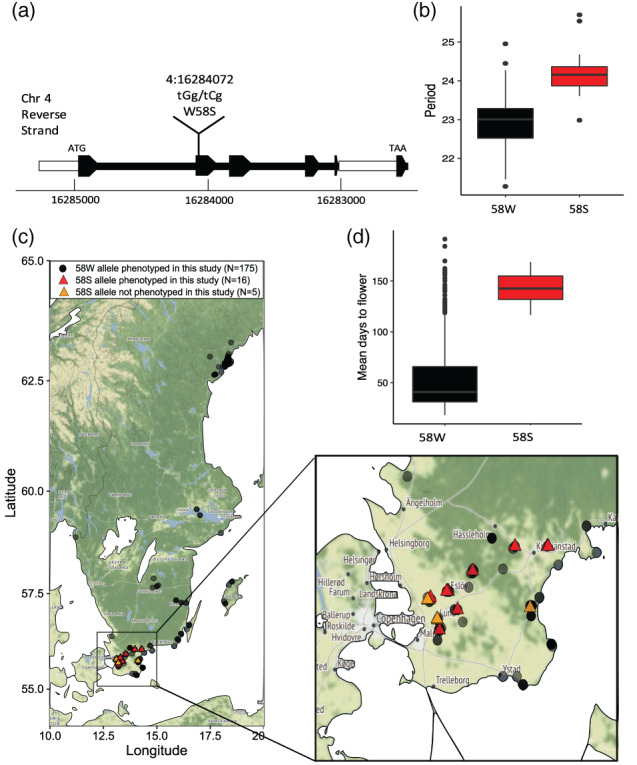
Natural variation in *COR28* is associated with covariation in period and flowering time. The SNP in *COR28* associated with period variation causes a tryptophan (W) to serine (S) amino acid change within the second exon (a). Accessions carrying the 58S allele had periods 1.29 hr longer on average than those with the 58W allele based on the 191‐accession data from this study (b). Out of the 191 accessions originally phenotyped, 16 had the 58S allele (red triangular points). Globally, 5 further 58S accessions were identified (orange triangles) and all were found in the south of Sweden (c). Accessions carrying the 58S allele also had flowering times delayed by 89.23 days compared to those carrying the 58W allele (data shown is based on flowering times from plants grown under Swedish conditions previously published in Li et al., 2010 (d) [Colour figure can be viewed at wileyonlinelibrary.com]

GWA identified multiple genomic regions associated with period, phase and RAE as shown in Figure 3. We used −log_10_(*p*) 6.5 as an arbitrary *p*‐score cut‐off to select SNPs for further investigation. This threshold is conservative compared to several other previously published studies in *Arabidopsis* (Kooke et al., 2016; Proietti et al., 2018; van Rooijen, Aarts, & Harbinson, 2015). We investigated known core circadian and flowering time genes to see whether these were significantly associated with any of the traits measured. Other than *ELF3* (see below), none of these genes fell within the window of association for significant SNPs. Genes in regions 30kb upstream or downstream of the most significant SNPs were considered as potential gene candidates and were selected for further analysis based on their previously attributed functionality and designated GO term. Genes involved in circadian rhythms, flowering time or chloroplast regulation were given prevalence (details in Supplementary File 2). The most significant associations had three SNPs with a −log_10_(*p*) score of 10.4–11.5, found on chromosome 4 associated with period variation. Within this interval we identified a non‐synonymous SNP in the gene *COLD‐REGULATED GENE 28*, a gene that has previously been identified as a negative regulator of several core clock genes (*PRR7*, *TOC1*, *PRR5* and *ELF4*) and is also implicated in the trade‐off between flowering‐time and freezing tolerance (Li et al., 2016; Wang et al., 2017). The substitution resulted in a tryptophan (W) to serine (S) amino acid change at position 58 within the second exon of *COR28* (Figure 4a). This had a SIFT score of 0 indicating a highly probable deleterious effect on protein function. Sixteen accessions in this study had the minor allele, all found in the South of Sweden. The 58S accessions had a period 1.29 hr longer than the 58W accessions (*t*(17.4) = −7.46, *p <* .001, Welch Two Sample *t* test) (see Figure 4b) and mostly belonged to the genomic sub‐group PC.A. The long‐period of cluster PC.C therefore cannot be explained by variation in this SNP. We used the online tool Polymorph 1001 to look for other variants with the serine substitution and found only five other variants not assayed in this study (making 21 in total), all of which were also from the South of Sweden (Figure 4c).

We used flowering time data by Li et al. (2010) and Sasaki et al. (2015) to look for flowering time differences between the accessions carrying the *COR28*‐58W major allele and the ‐58S minor allele (see Table 1). We found that the accessions with *COR28*‐58S had significantly extended flowering times under simulated seasons for Sweden and Spain (Li) and under a 10°C and 16°C long‐day temperature regime (Sasaki) (see Figure 4d). This complements previous findings that *COR28* is a flowering promoter and that modification to this gene increase flowering time as well as lengthening period.

**TABLE 1:** Longer flowering time correlation with *COR28* W58S Allele

Datasets				Days to flower		Welch Two sample *t* test		
		*N* for 58W	*N* for 58S	Mean 58W	Mean 58S	d.f.	*t*	*p*
Li	Sweden average	470	10	54.43	143.66	10.8	16.46	<0.001
	Spain average			64.78	122.67	9.5	9.70	<0.001
Sasaki	10°C	1145	18	83.24	100.92	17.8	4.93	<0.001
	16°C			73.97	94.91	17.6	5.75	<0.001

*Notes*: *N* = number of accessions used for each *COR28* allele from a global population. Flowering data means reflect an average of all replicates within each study by Li et al. (2010) or Sasaki et al. (2015).

Other significant SNPs associated with period (chromosome 3) and phase (chromosome 1) identified gene candidates involved in chloroplast function (*PARC6*, *SCO2*, *DNF*, *AT1G71015* and *FAB1C*). The DF circadian output we used to assay these traits is based on oscillating activation phases of PSII (Goltsev, Zaharieva, Chernev, & R, 2009; Jursinic, 1986) and therefore natural variation in genes regulating chloroplast functionally could also affect the DF output in these candidates.

A RAE associated SNP on chromosome 2 was found 5726bp upstream from a SNP in *ELF3* previously characterized as the *ELF3‐Sha* allele. Our associated SNP was found to be under strong linkage disequilibrium with the *ELF3‐Sha* allele (*R* ^2^ = 0.86). The alanine‐to‐valine transition in amino acid position 362 has been associated with naturally occurring alterations to periodicity and robustness in accessions from Central Asia, specifically Tajikistan (Anwer et al., 2014). Here, 13 Swedish accessions were shown to carry the *ELF‐Sha* allele and had mean RAE ratios 0.032 higher on average than for the other accessions. We substantiate evidence that *ELF3‐Sha* accessions have lower rhythmicity and extend the global range of this allele into Northern Sweden. A surprising kinship between *Arabidopsis* accessions from Northern Sweden and Central Asia has been previously demonstrated through analysis of global population structure and indicates that the presence of this allele has not evolved convergently between the two populations (Nordborg et al., 2005).

### 3.3 Validation of *COR28* effects using knockout lines and co‐segregation analysis

Eleven mutant lines representing eight gene candidates were genotyped to confirm homozygous mutations before being bulked for seed. Confirmed mutants were then assayed for their circadian rhythms using DF under the same conditions as for the 191 accession screen. Mutant details and genotyping results can be viewed in Supplementary File 2. *Cor27* mutants and the double mutant *cor27‐1/28‐2* were also assayed as *cor27* is known to be partially redundant with *cor28*. Analysis of the DF rhythms confirmed that periods in *cor28* mutants (*cor28‐1* = 25.3 hr, *SD* = 1.73; *cor28‐2* = 25.3 hr, *SD* = 1.78) were significantly longer than their respective controls (23.9 hr, *SD* = 1.62 and 24.0 hr, *SD* = 2.02) (Welch Two Sample *t* test *p <* .05) as shown in Table 2. This confirms results previously reported using leaf movement, qPCR and ProCCR2:LUC bioluminescence rhythms (Li et al., 2016; Wang et al., 2017). *Cor27* mutants showed no significant difference in period length, however the double knock‐out *cor27‐1/28‐2* had an exaggerated long period phenotype (26.4 hr, *SD* = 2.37). The peak phases of *cor27*, *cor28* and especially *cor27‐1/28‐2* were also earlier than for Col‐0, with *cor27‐1/28‐2* having a mean phase peak 2.6 hr earlier than the control. Other mutants characterized were not found to have significantly altered periods or phases compared to their WT controls (Tables S12–S14).

**TABLE 2:** Mean results from DF screening of GWA candidate mutant lines

Plant ID	*N*	Period mean (hr)	Period *SD*	RAE mean	RAE *SD*	Phase circular mean (hr)	Phase *SD*
**col‐0**	**180**	**24**	**2.02**	**0.42**	**0.18**	**15.35**	**1.04**
*cor27‐1*	108	24	1.75	0.34	0.18	13.39	0.77
*cor27‐2*	107	24.2	2.21	0.29	0.14	14.02	0.83
*cor28‐2*	158	25.3	1.78	0.28	0.13	12.93	0.91
*cor28‐2/27‐1*	101	26.5	2.33	0.28	0.14	12.67	0.98
*mybl2*	103	24	1.97	0.37	0.17	14.27	0.93
*parc6*	97	23.9	2.08	0.37	0.16	13.91	0.97
*parc6‐1*	104	23.4	1.77	0.35	0.17	14.95	0.87
*atg1g71015*	102	23.7	2.11	0.38	0.16	14.45	0.92
**WT for *cor28‐1***	**72**	**23.9**	**1.62**	**0.33**	**0.17**	**14.54**	**0.79**
*cor28‐1*	70	25.3	1.73	0.28	0.16	12.95	0.86
**ler**	**108**	**23.2**	**2.55**	**0.43**	**0.17**	**16.37**	**1.04**
*sco2*	101	23.7	1.95	0.51	0.16	15.33	1.00

*Notes*: Mutants are listed under their respective WT controls in bold. Circular means for phase were calculated using the “circular” package in R.

Finally, we carried out a co‐segregation analysis for the *COR28* 58S allele to demonstrate that this SNP was causal for the long period phenotype. Crosses were made between the line T800 (Watkins 6133) and St‐0 (Watkins 8387), with T800 carrying the 58S minor allele and St‐0 carrying the 58W major allele. Parent individuals were genotyped using a CAPs assay prior to crossing and were found to be homozygous for their respective SNPs. We selfed three F_1_ individuals from this cross and analysed the genotypes and circadian period phenotypes in the F_2_ offspring using DF on dissected leaves (Table 3). Restriction digest gels from F_2_ genotyping can be viewed in Supplementary File 6. F_2_ individuals carrying the 58S SNP had a mean period length of 23.7 hr (*SD* = 1.95) compared to a mean of 21.3 hr (*SD* = 1.65) for those carrying the 58W SNP (a difference of 2.4 hr) which was shown to be highly significant (*F*(2,62) = 12.27, *p* < .0001, one‐way ANOVA. Tukey HSD for SNP 58S and 58W: *p* < .001). Heterozygous individuals had period lengths which were on average closer to the long period phenotypes of the 58S homozygote individuals (23.1 hr (*SD* = 1.95)) suggesting a semi‐dominant effect for the 58S allele.

**TABLE 3:** Results from co‐segregation analysis of the *COR28* SNP 58S with long period

	Genotyping							Phenotyping		
F_1_ ID	Genotype	*N* plants genotyped	Expected ratio of genotypes	d.f.	*χ* ^2^	*p* value	Mean period (hr)	SD period	*F* value	*p* value
*T1A1*	*HZ*	9	10.5	2	0.43	0.81	22.09	1.139	26.55	<.00001***
	*HM 58S*	6	5.25	2			24.38	0.902		
	*HM 58W*	6	5.25	2			20.50	0.469		
*T1A2*	*HZ*	17	13	2	3.15	0.20	23.75	1.92	1.826	0.184
	*HM 58S*	3	6.5	2			22.51	3.33		
	*HM 58W*	6	6.5	2			21.96	1.92		
*T1A3*	*HZ*	14	13.5	2	3.67	0.16	23.05	1.043	4.08	0.0299*
	*HM 58S*	3	6.75	2			23.58	2.050		
	*HM 58W*	10	6.75	2			21.47	1.856		

*Notes*: Three heterozygous F_1_ plants were selfed to produce F_2_ offspring broadly following a Mendelain segregation ratio of 2:1:1 tested using a Chi‐Square test. F_2_ individuals were phenotyped using DF imaging on detached leaves and were analysed within each F_1_ group using a one‐way ANOVA (analysis across all F_1_ individuals presented in main text). *p < 0.05; ***p < 0.001.

### 3.4 The phenotypic tails of circadian traits remain largely diverged under lower temperatures

The variation characterized in the first part of this paper was observed at 22°C which was chosen to make the data comparable to several previously published studies of interest. We wanted to test whether the circadian phenotypic diversity we observed at 22°C continued at temperatures of 16°C and 10°C; closer to those found in the natural Swedish environment. To simplify the dataset, we selected 10 accessions to represent each of the six phenotypic tails as highlighted in Figure 1. We wished to investigate whether reduced temperatures would affect the phenotypic tails equally, drive them further apart or lead to a convergence of their phenotypes. Our null hypothesis was that the phenotypic diversity seen at 22°C would exist consistently at lower temperatures with no differential effect on the phenotypic tail groups.

**FIGURE 5:**
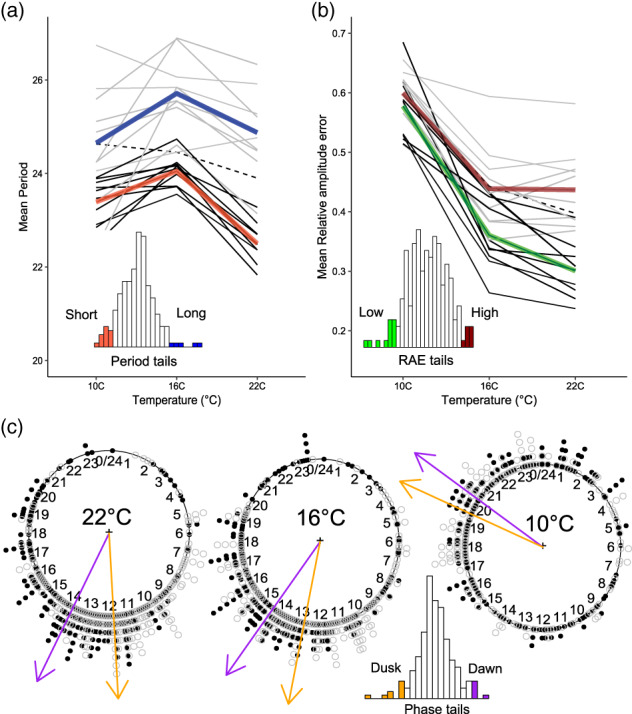
Effects of temperature on circadian rhythms in phenotypic tails. We selected 10 accessions to represent the phenotypic tails of each trait as highlighted in Figure 1. Period, phase and RAE in these accessions were then re‐quantified at 10°C, 16°C and 22°C to see if the tails remained diverged at lower temperatures. In figure (a), light grey lines show the mean periods of accessions in the long period tail group with the thick blue line showing the mean period for the whole group. Black lines show the mean periods for accessions in the short period group with the thick red line reflecting the mean for the whole short period group. In figure (b), light grey lines show the mean periods of accessions with high RAE (low robustness) with the thick maroon line indicating the overall high RAE tail mean. The black lines show the mean periods for accessions with low RAE (high robustness). The green line shows the overall low RAE tail mean. In both figures (a) and (b) the dotted line is the mean for Col‐0 at each temperature. Figure (c) shows individual phase estimates from accessions belonging to the dusk‐phased (dark filled point) or dawn‐phased (open point) tail groups plotted as clock plots relative to 24/0 representing dawn. Tail group phase means are indicated by coloured arrows (dusk tail in orange and dawn tail in purple). Sample size contributing to accession mean at each temperature ranged from 14 to 24 [Colour figure can be viewed at wileyonlinelibrary.com]

The results show that the decreasing temperature had a massive overall effect on all three circadian outputs, particularly RAE and phase (accession means can be viewed in Supplementary File 3). Overall, the divergence between the tail groups was largely maintained, although the gap reduced at 10°C (Figure 5a–c). For each trait, a general linear model was fitted to the data in order to test the significance of explanatory factors (Tables S15–S17).

For period, membership to the short or long tail groups was the largest explanatory variable and the group means remained clearly distinct across all temperatures. Temperature also had a large overall effect on period, with rhythms at 16°C running much slower than at 22°C. At 10°C periods were again shorter, accompanied by higher RAE (reduced rhythm robustness) (Figure S7). The difference in the period temperature responses of the two groups can be seen by the gradients of the thick coloured lines in Figure 5a. There was also significant variation between the temperature response of individual accessions within each tail group, especially in the long period group (see thin grey lines in Figure 5a). Interestingly, Col‐0 reacted very differently to the Swedish accessions tested, showing an almost linear decrease in period with increasing temperature (dashed line in Figure 5a).

For RAE, which we equate to rhythm robustness, we found that temperature had an even greater effect than for period, with rhythms at 10°C showing a marked decrease in robustness (Figure 5b). The tails converge as the temperature decreases, with lower temperatures having an especially large effect on the low RAE group.

For phase, decreasing temperature to 10°C caused a large shift of approximately 7.4 hr towards dawn accompanied by increased variability for each accession (Figure 5c). The means of the two phase‐tail groups remained distinct across the three temperatures and there was no significant difference in their relative change of phase with temperature.

We also conducted an independent experiment with the phase tail accessions to verify the phase estimates from the two seed batches measured at 22°C (Figure S9).

Across all traits we observed an increase in the within‐accession variability at lower temperatures indicated by larger standard deviations in period, higher RAE scores and a greater number of rhythms being rejected from Biodare2 analysis as arrhythmic.

## 4 DISCUSSION

We measured DF rhythms in 191 naturally occurring Swedish *Arabidopsis* accessions and show that circadian phenotypes display considerable variation. This variation does not conform to previously described latitudinal clines in *Arabidopsis* (Michael et al., 2003), as we find the longest periods in accessions from the South of the country and the shortest periods in the North. In our study, circadian phenotypes are also clustered into geographical groups rather than following a distinct latitudinal cline. We suggest that the period variation we observe in the Swedish population is due largely to founder effects from ancestral migrations of individuals adapted to different selection pressures (Flohr, Blom, Rainey, & Beaumont, 2013), and may be influenced by serendipitous fixing of alleles through genetic drift (Song, Dey, & Holsinger, 2006).

We found a high level of correlation between circadian period, phase and RAE in these accessions; period was negatively correlated with both RAE and phase. Interestingly, in the study by Michael et al. (2003), period, phase and amplitude were reported to vary independently for leaf movement rhythms in a global panel.

Periods were found to be significantly different between two sub‐populations co‐existing in the same geographical area in the South. In spite of this, accessions with very long periods (belonging to the long‐period phenotypic tail) were found in both of these populations and the minor *COR28* SNP was also found in both the PC.A and PC.C sub‐groups.

As noted in the results, the correlation of period and phase with flowering time appears to be highly dependent on the experimental conditions under which flowering time is approximated. We hypothesize that this also applies to the conditions under which circadian rhythms are measured. It would be particularly interesting to see how this correlation changes when rhythms are assayed in more mature plants close to flowering transition, as period length is known to be greatly affected by developmental stage (Kim, Kim, Yeom, Lim, & Nam, 2016; Rees et al., 2019).

We show that circadian variation assayed by DF is genetically heritable and is associated with several highly significant polymorphisms, two of which (*ELF3* and *COR28*) had previously acknowledged circadian functions. *COR28* is partially redundant with its partner *COR27* and acts both upstream and downstream of the circadian clock (Li et al., 2016; Wang et al., 2017). TDNA insertions in these genes have been shown to lengthen periods, extend flowering time and increase freezing tolerance (Li et al., 2016; Wang et al., 2017). Natural allelic diversity in *COR28* has not previously been described. Here, we show that a set of 16 naturally occurring accessions from southern Sweden have a W58S amino acid substitution which results in a long period comparable to that seen in *cor28* TDNA insert mutants. *COR28* and *COR27* are expressed in a blue‐light and temperature dependent manner, plausibly suggesting why this gene could be under selection in the Swedish environment. The mechanism through which W58S affects the function of COR28 remains unclear. COR28 is a small peptide of ~26 kDa and so may not require active transport for nuclear localization (Hicks, 2013). No DNA binding domains have been identified in the COR28 sequence, however, it has been suggested to regulate *TOC1* and *PRR5* transcription through the formation of protein complexes (Li et al., 2016). It is possible that this modification affects the ability of COR28 to form these protein interactions.

We also identified ELF‐sha alleles in Northern accessions which were associated with higher RAE ratios. In barley, a mutant ortholog of *ELF3*; *eam8* was shown to have been positively selected for growth in high‐latitude environments, particularly in Scandinavia (Zakhrabekova et al., 2012). *eam8* cultivars are rapid flowering enabling survival under short growing seasons, but also have severally attenuated circadian function (Faure et al., 2012).

In addition to the loci supporting *COR28* and *ELF3* association with circadian variation, two other peaks passed the −log_10_(*p*) threshold associated with phase and period length. TDNA mutants for candidate genes in these regions did not display significantly altered circadian rhythms, however it is possible that these genes may well have a genuine effect on circadian function not detected in the knock‐out line. These associated SNPs may be causing an alteration or gain of function which would be missed by bluntly knocking out the gene with a TDNA insert. It is also likely that the associated SNPs in our Manhattan plots are in linkage disequilibrium with genes which were not screened as potential gene candidates within this study.

Finally, we investigated the effect of temperature on natural circadian variation between accessions with divergent circadian phenotypes. Temperature had a large effect on period and an even greater one on phase and RAE means in both tail groups, indicating a low level of temperature compensation for these outputs. This also shows that the forces governing compensation for period do not act equally on maintaining constant RAE or peak phase. Interestingly, rhythms appeared to be most robust at 22°C, which might not be expected given that the warmest months in Sweden average around 15–17°C. A similar loss of rhythm robustness at lower temperatures has been observed in wheat (Rees et al., 2019).

Period had a non‐linear relationship with temperature in these accessions, lengthening from 22 to 16°C, before shortening again at 10°C. This arrow shaped profile likely reflects two interacting forces at work: (a) between 22 and 27°C the acceleration of rhythms due to increased rate kinetics and (2) between 22 and 10°C the balancing forces of circadian temperature compensation. Gould et al. found a similar effect of temperature on period using leaf‐movement rhythms at 22, 17 and 12°C, and showed that the cold temperature compensation response works through an independent mechanism to the hot temperature compensation response (Gould et al., 2006). The profile suggests that temperature compensation is biased towards correction at colder temperatures in these accessions; at least for the DF circadian output. It is possible that adaptation to a cold climate has selected for a cold compensation response to overcome excessive deceleration of the clock, although we are unable to explain why the rhythms should be even shorter at 10°C than at 16°C or why the shortening of periods are accompanied by a loss of overall rhythmicity. Although divergence between the phenotypic tails decreased at lower temperatures, the groups remained largely separate, reconfirming that these tail phenotypes are due to heritable genotypic differences. This work demonstrates the utility of using DF imaging to analyse natural variation across genetically diverse populations.

## AUTHOR CONTRIBUTIONS

This project was conceptualized by Anthony Hall and Hannah Rees. Hannah Rees designed and conducted imaging experiments and mutant screening. James K. M. Brown and Hannah Rees carried out statistical analysis and data processing. The GWA analysis was done by Ryan Joynson and Hannah Rees. All authors contributed to the interpretation of results. The paper was written by Hannah Rees with assistance from Ryan Joynson, James K. M. Brown and Anthony Hall. All authors approved the final manuscript.

## Supporting information


**Appendix** **S1**: Supporting Information

**Figure S1**: Genstat output, residual plots to check for normality and outliers in period data from 191 accessions (191 accession data)
**Figure S2**: Genstat output, residual plots to check for normality and outliers in RAE data from 191 accessions (191 accession data)
**Figure S3**: Genstat output, residual plots to check for normality and outliers in Log_10_Amp data from 191 accessions
**Figure S4**: Correlations between REML adjusted accession means for period, phase and RAE for each accession in the 191 accession dataset
**Figure S5**: Period correlation with latitude and longitude (191 accession data)
**Figure S6**: Manhattan plots and Q‐Q plots from all GWA models (191 accession data)
**Figure S7**: Period and RAE correlations in period tail accessions (temperature data)
**Figure S8**: Position in 96‐well plate affects period estimation‐justification for removing these wells from analysis (191 accession data).
**Figure S9**: Verification of phase predictions at 22°C using two seed batches
**Table S1**: Output from Genstat using the REML directive on 191 period data
**Table S2**: Output from Genstat using the REML directive on 191 RAE data
**Table S3**: Output from Genstat using the REML directive on 191 log_10_Amplitude data
**Table S4**: Likelihood test for period (191 accession data)
**Table S5**: Likelihood test for phase (191 accession data)
**Table S6**: Likelihood test for RAE (191 accession data)
**Table S7**: Likelihood test for Log_10_Amp (191 accession data)
**Table S8**: Accessions in period tails for temperature experiments (temperature data)
**Table S9**: Accessions in phase tails for temperature experiments (temperature data)
**Table S10**: Accessions in RAE Tails for temperature experiments (temperature data)
**Table S11**: Linear regression with previously published datasets (191 accession data)
**Table S12**: Testing differences in period (Welch Two Sample t‐test) (Mutant validation data)
**Table S13**: Testing differences in RAE (Welch Two Sample *t* test) (mutant validation data)
**Table S14**: Testing differences in phase (Watson's Two‐Sample Test of Homogeneity) (mutant validation data)
**Table S15**: Accumulated analysis of variance table for period with temperature data
**Table S16**: Accumulated analysis of variance table for RAE with temperature data
**Table S17**: Circular regression analysis for phase with temperature data

**Supplementary File 1** Phenotype files from 191 dataset

**Supplementary File 2** Gene candidates for DF phenotyping

**Supplementary File 3** Line means and *SD* for temperature experiments

**Supplementary File 4** Generate_all_rois_96_plate

**Supplementary File 5** Selecting ROI for DF imaging using multiple 96‐well plates

**Supplementary File 6** Gels from CAPS assay for co‐segregation analysis
