# Supplementary material for: Naturally occurring circadian rhythm variation associated with clock gene loci in Swedish Arabidopsis accessions
Source: Plant Cell Environ. 2021 Jan 11;44(3):807–20. doi: 10.1111/pce.13941 (PMC7986795; doi:10.1111/pce.13941)
Supplement: Supplementary file 2 — Figure S1: Genstat output, residual plots to check for normality and outliers in period data from 191 accessions (191 accession data) Figure S2: Genstat output, residual plots to check for normality and outliers in RAE data from 191 accessions (191 accession data) Figure S3: Genstat output, residual plots to check for normality and outliers in Log10Amp data from 191 accessions Figure S4: Correlations between REML adjusted accession means for period, phase and RAE for each accession in the 191 accession dataset Figure S5: Period correlation with latitude and longitude (191 accession data) Figure S6: Manhattan plots and Q‐Q plots from all GWA models (191 accession data) Figure S7: Period and RAE correlations in period tail accessions (temperature data) Figure S8: Position in 96‐well plate affects period estimation‐justification for removing these wells from analysis (191 accession data). Figure S9: Verification of phase predictions at 22°C using two seed batches Table S1: Output from Genstat using the REML directive on 191 period data Table S2: Output from Genstat using the REML directive on 191 RAE data Table S3: Output from Genstat using the REML directive on 191 log10Amplitude data Table S4: Likelihood test for period (191 accession data) Table S5: Likelihood test for phase (191 accession data) Table S6: Likelihood test for RAE (191 accession data) Table S7: Likelihood test for Log10Amp (191 accession data) Table S8: Accessions in period tails for temperature experiments (temperature data) Table S9: Accessions in phase tails for temperature experiments (temperature data) Table S10: Accessions in RAE Tails for temperature experiments (temperature data) Table S11: Linear regression with previously published datasets (191 accession data) Table S12: Testing differences in period (Welch Two Sample t‐test) (Mutant validation data) Table S13: Testing differences in RAE (Welch Two Sample t test) (mutant validation data) Table S14: Testing differences in phase (Watson's Two [file PCE-44-807-s008.pdf]

Supplementary Figures and Tables:

Supplementary Figure 1	Genstat output, residual Plots to check for normality and outliers in period data from 191 accessions (191 accession data)
Supplementary Figure 2	Genstat output, residual Plots to check for normality and outliers in RAE data from 191 accessions (191 accession data)
Supplementary Figure 3	Genstat output, residual Plots to check for normality and outliers in Log10Amp data from 191 accessions
Supplementary Figure 4.	Correlations between REML adjusted accession means for Period, Phase and RAE for each accession in the 191 accession dataset.
Supplementary Figure 5	Period correlation with latitude and longitude (191 accession data)
Supplementary Figure 6	Manhattan plots and Q-Q plots from all GWA models (191 accession data)
Supplementary Figure 7	Period and RAE correlations in Period tail accessions (Temperature data)
Supplementary Figure 8	Position in 96-well plate affects period estimation-justification for removing these wells from analysis (191 accession data).
Supplementary Figure 9	Verification of phase predictions at 22C using two seed batches
Supplementary Table 1	Output from Genstat using the REML directive on 191 period data
Supplementary Table 2	Output from Genstat using the REML directive on 191 RAE data
Supplementary Table 3	Output from Genstat using the REML directive on 191 log10Amplitude data
Supplementary Table 4	Likelihood test for period (191 accession data)
Supplementary Table 5	Likelihood test for phase (191 accession data)

Supplementary Table 6	Likelihood test for RAE (191 accession data)
Supplementary Table 7	Likelihood test for Log10Amp (191 accession data)
Supplementary Table 8	Accessions in Period Tails for temperature experiments (Temperature data)
Supplementary Table 9	Accessions in Phase Tails for temperature experiments (Temperature data)
Supplementary Table 10	Accessions in RAE Tails for temperature experiments (Temperature data)
Supplementary Table 11	Linear regression with previously published datasets (191 accession data)
Supplementary Table 12	Testing differences in Period (Welch Two Sample t-test) (Mutant validation data)
Supplementary Table 13	Supplementary Table S3: Testing differences in RAE (Welch Two Sample t-test) (Mutant validation data)
Supplementary Table 14	Supplementary Table S4: Testing differences in Phase (Watson's Two-Sample Test of Homogeneity) (Mutant validation data)
Supplementary Table 15	Accumulated analysis of varience table for period with temperature data
Supplementary Table 16	Accumulated analysis of varience table for RAE with temperature data
Supplementary Table 17	Circular regression analysis for Phase with temperature data

Model Checking: Period, RAE, Log10Amplitude

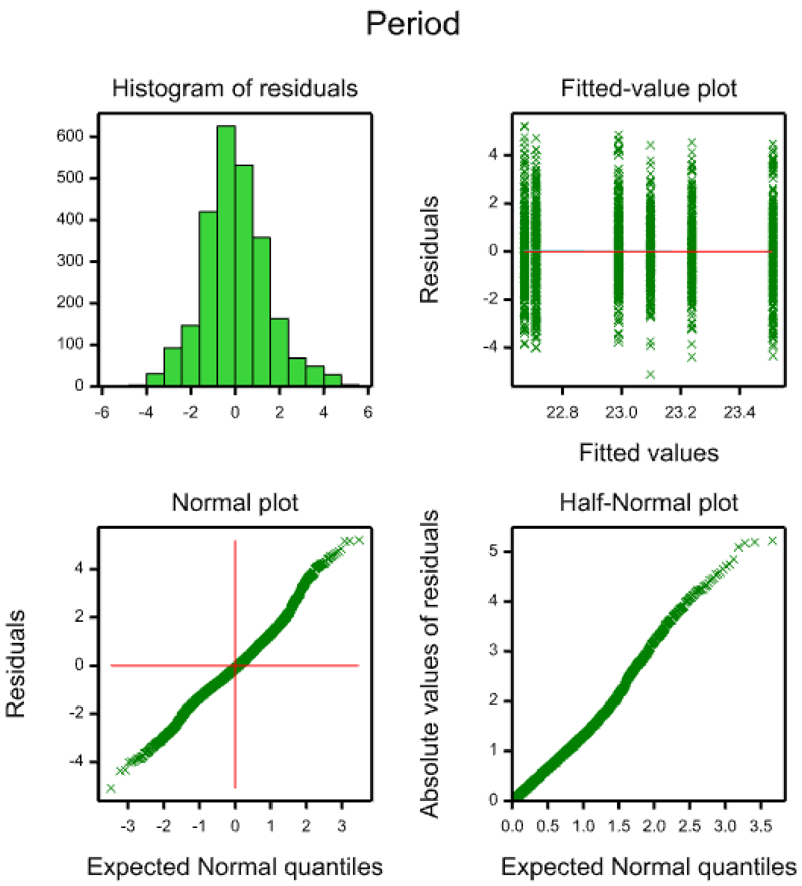

Supplementary Figure 1. Genstat output, residual Plots to check for normality and outliers in period data from 191 accessions

RAE

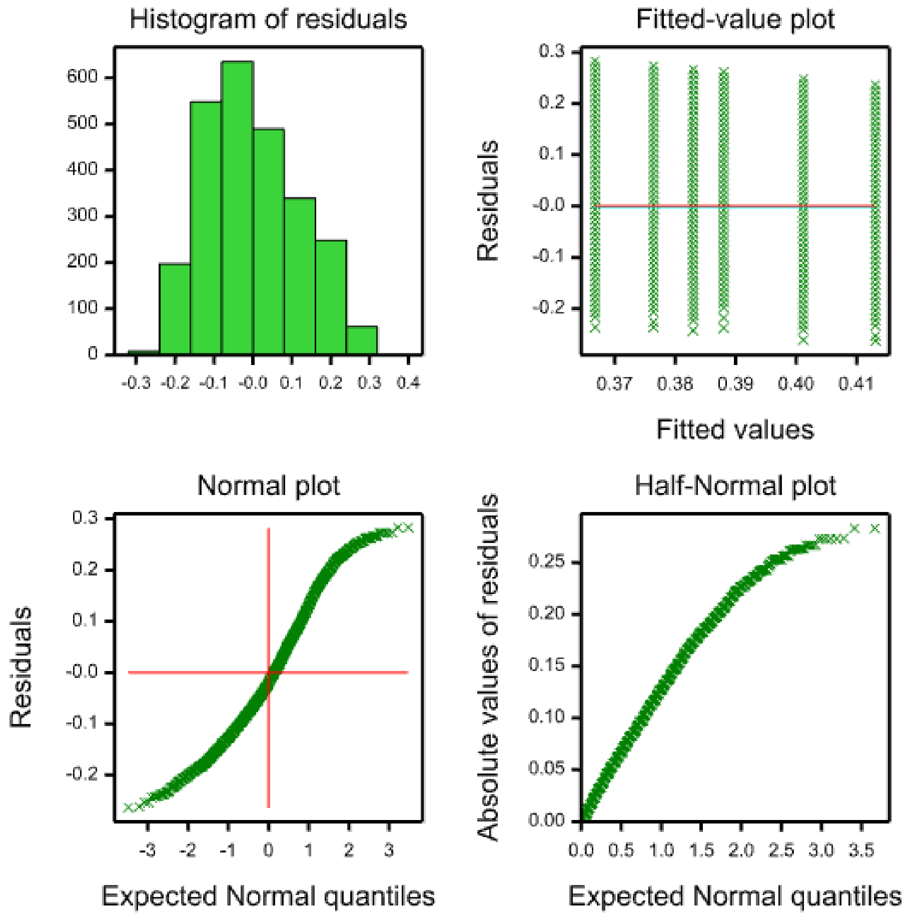

Supplementary Figure 2. Genstat output, residual Plots to check for normality and outliers in RAE data from 191 accessions

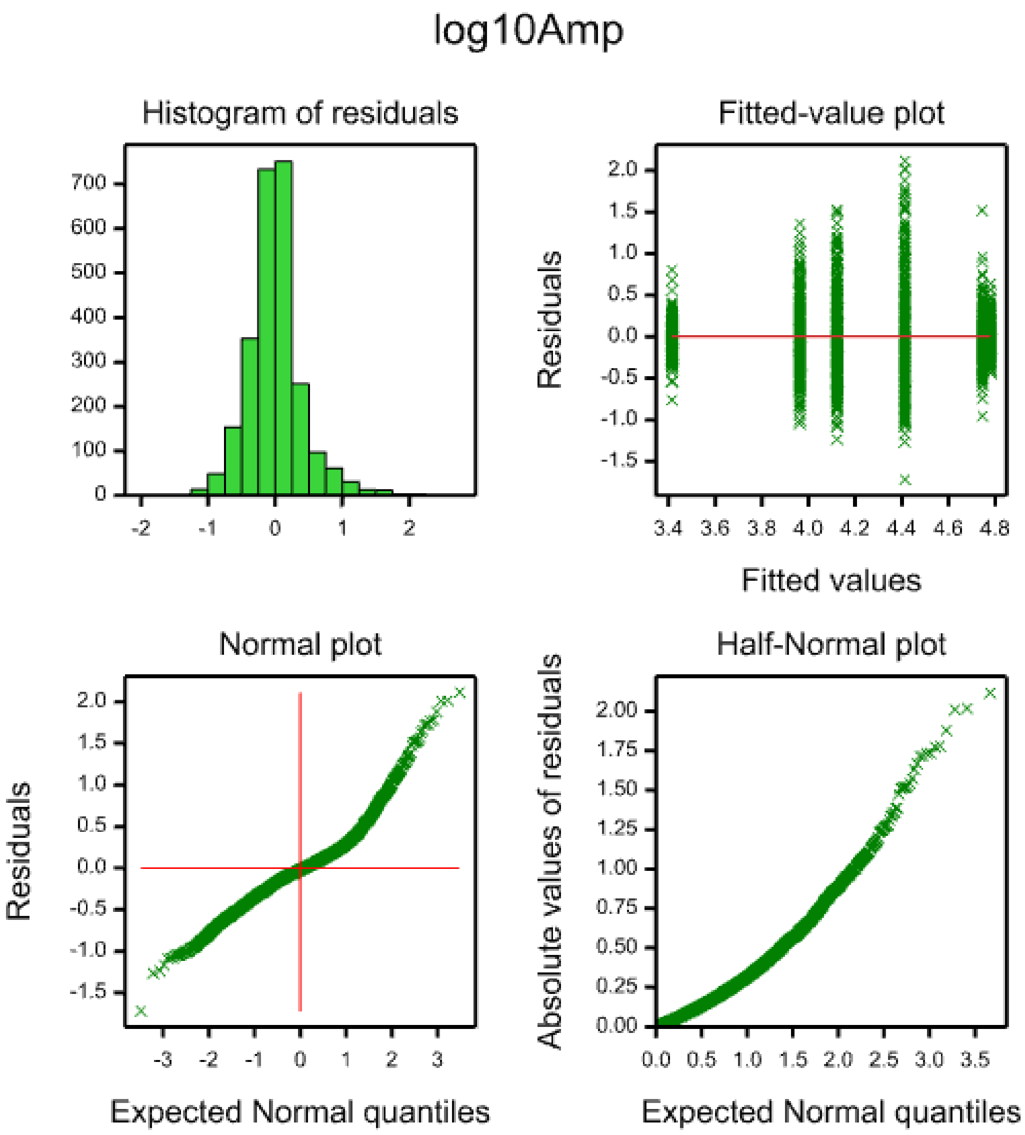

Supplementary Figure 3. Genstat output, residual Plots to check for normality and outliers in log10Amplitude data from 191 accessions

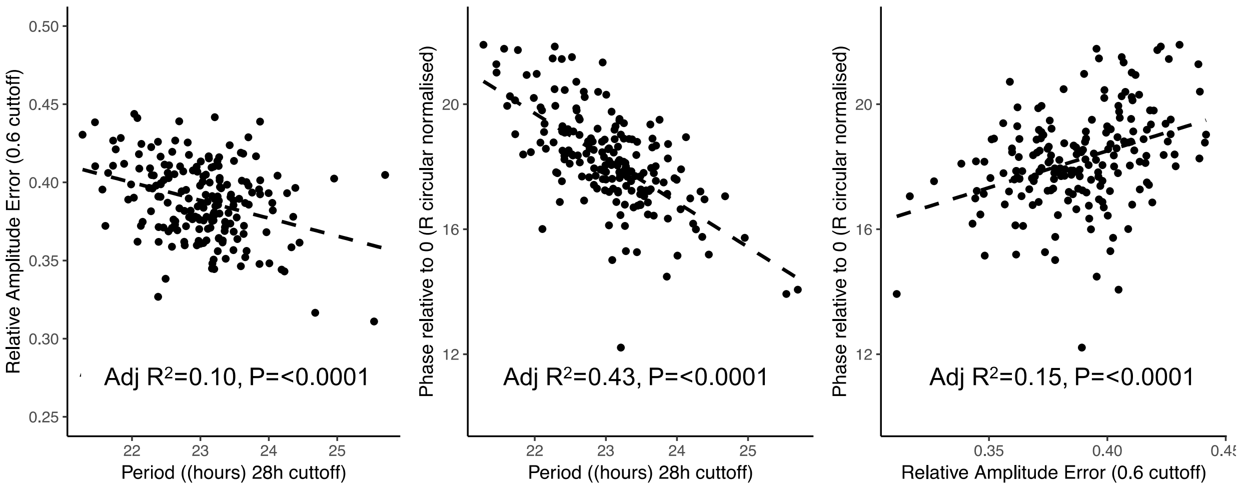

Supplementary Figure 4. Correlations between REML adjusted accession means for Period, Phase and RAE for each accession in the 191 accession dataset.

Adjusted R^2 and p-values were calculated using a linear model in R, on 1 and 189 degrees of freedom.

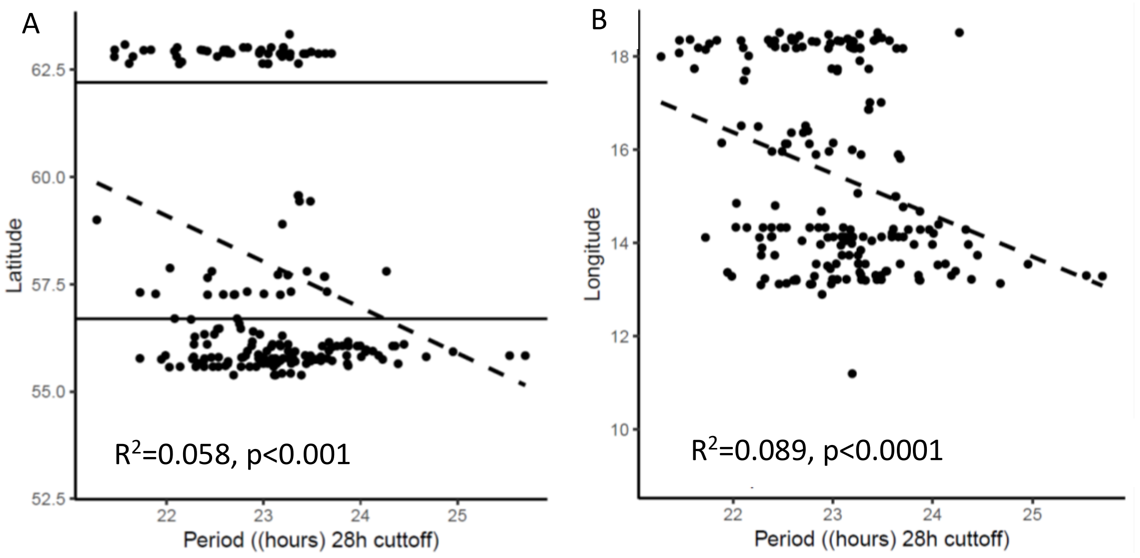

Supplementary Figure5. Period correlation with latitude and longitude
Period is significantly correlated with latitude (A). Horizontal lines differentiate between North, Mid and South Sweden. The dotted dashed line is a regression line. Period is significantly correlated with longitude (B).

Supplementary Figure 6. Manhattan plots and Q-Q plots from all GWA models (Downloaded from GWA-portal. Bonferroni (Red dotted line) and Benjamini Hochberg (Blue dotted line) thresholds were deemed too stringent and therefore we used an arbitrary -log10*p* value of 6.5 as a cut-off for further investigation (see main Figure 3).

Period

Linear model

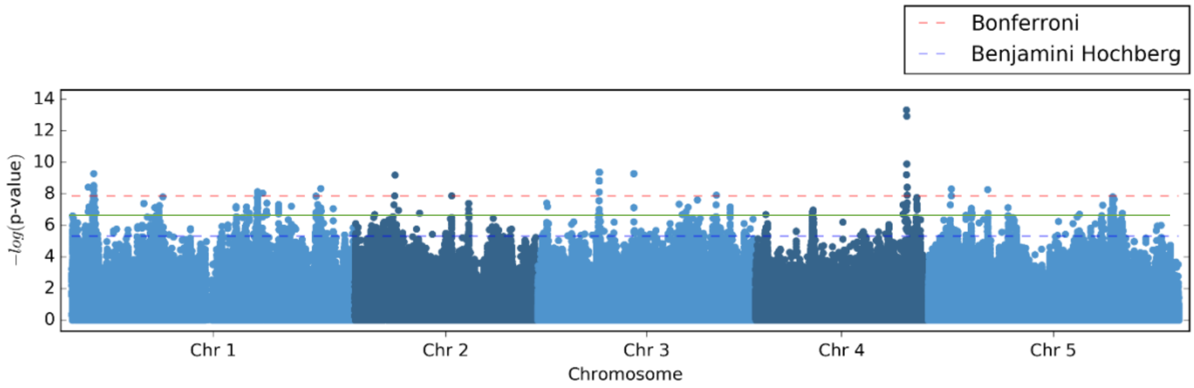

Kruskal-Wallis Model

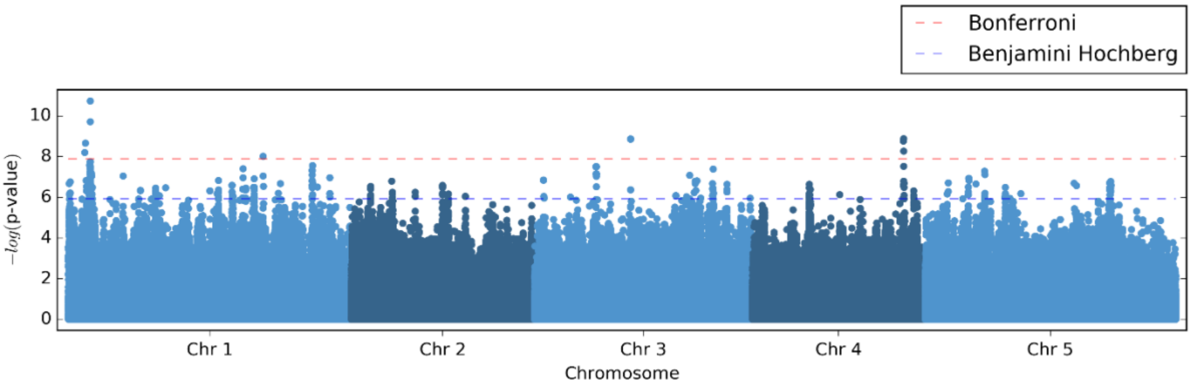

Accelerated Mixed Model

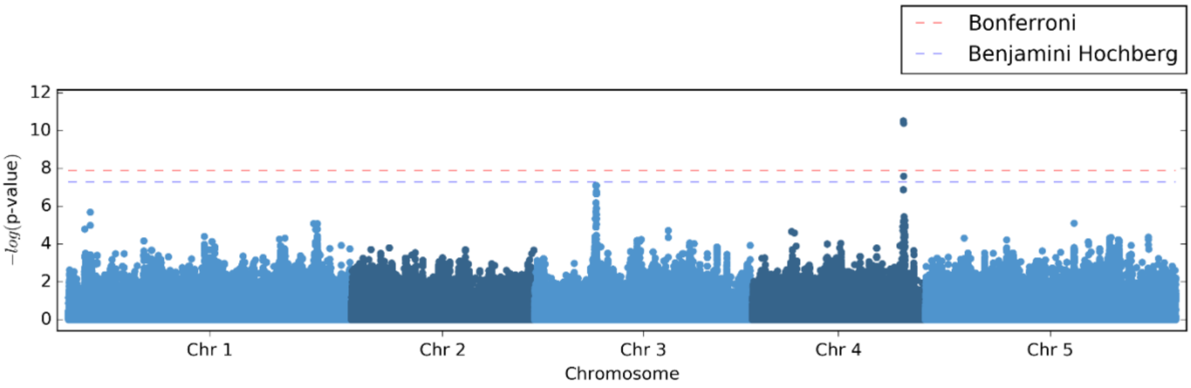

Linear model

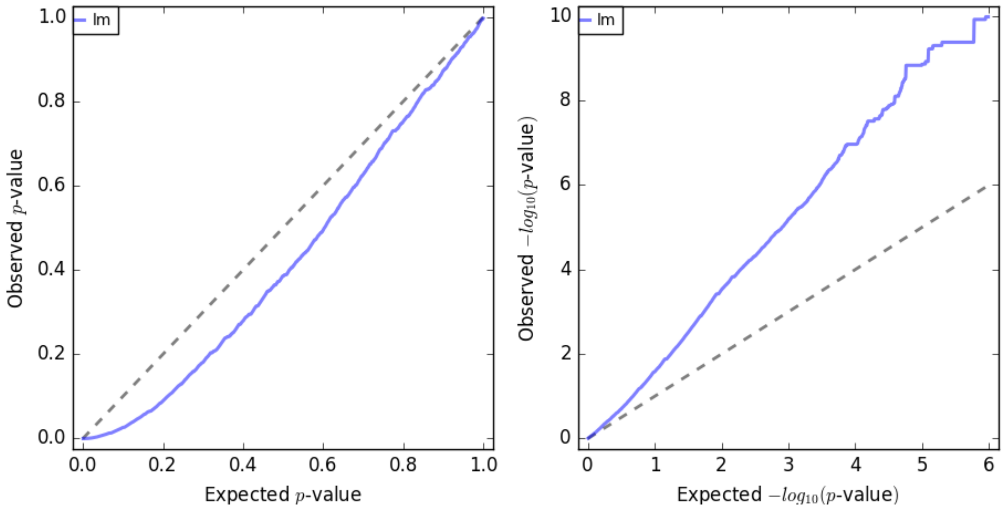

Kruskal-Wallis Model

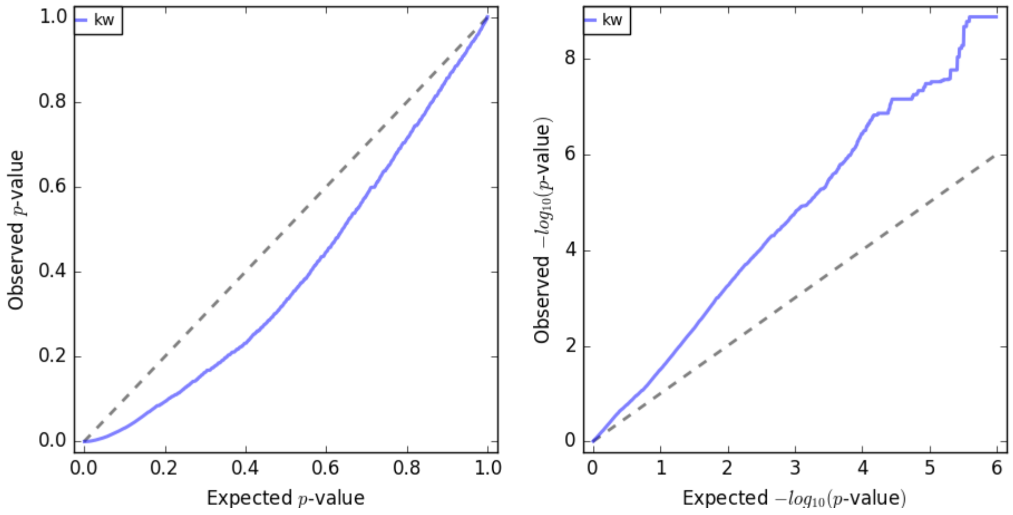

Accelerated Mixed Model

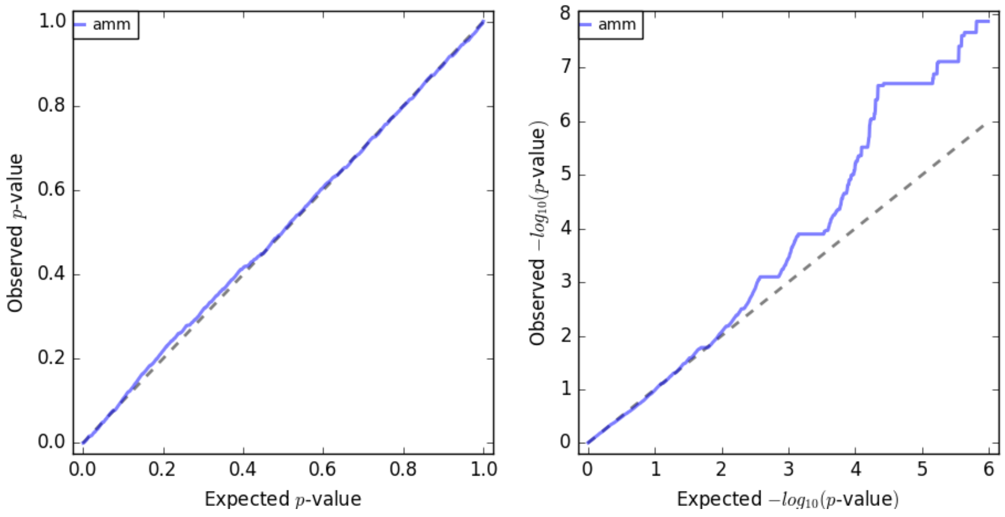

RAE

Linear model

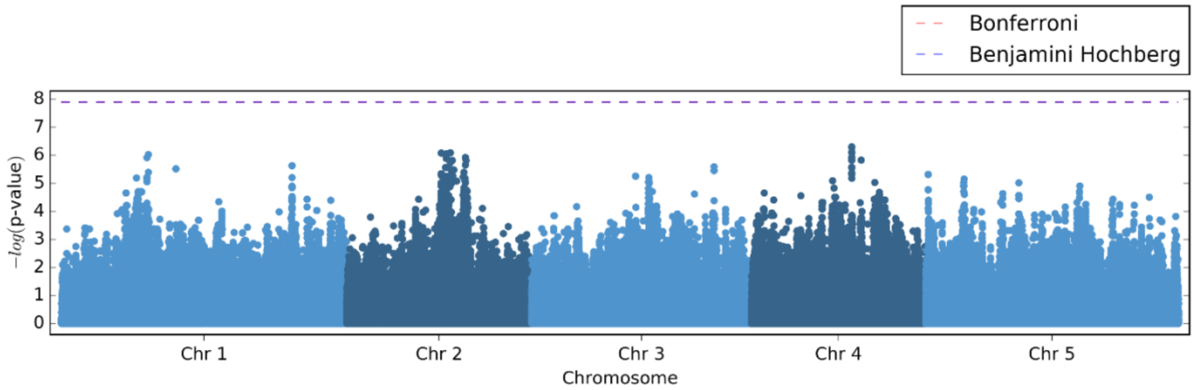

Kruskal-Wallis Model

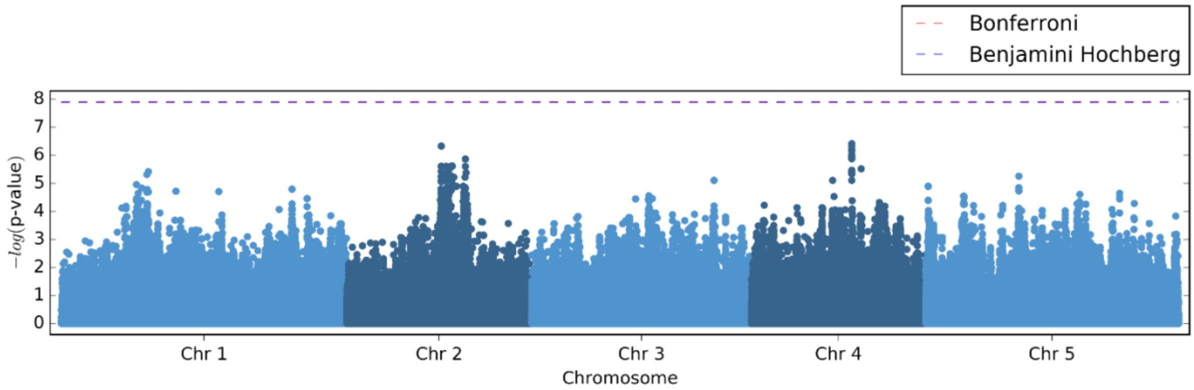

Accelerated Mixed Model

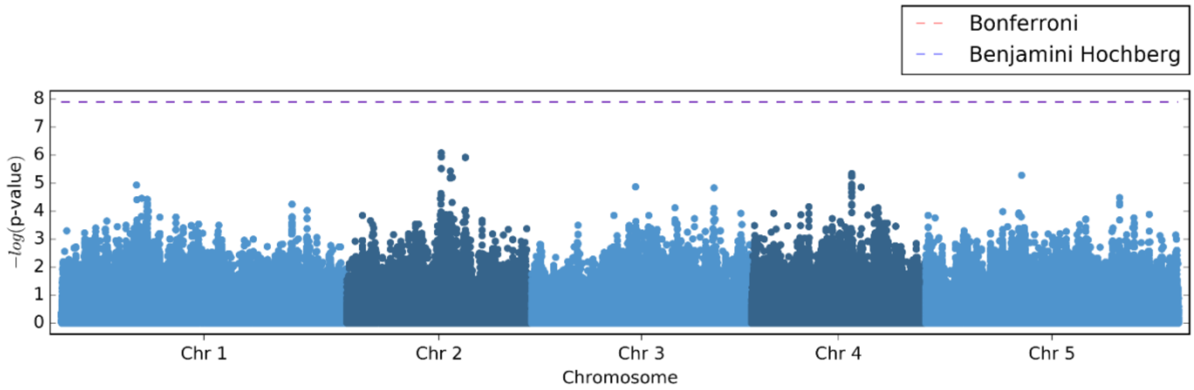

Linear model

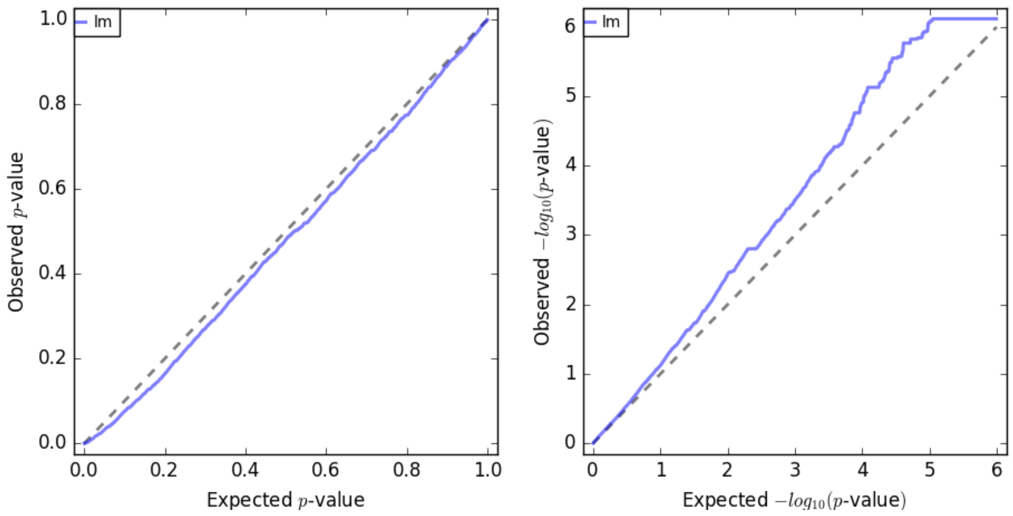

Kruskal-Wallis Model

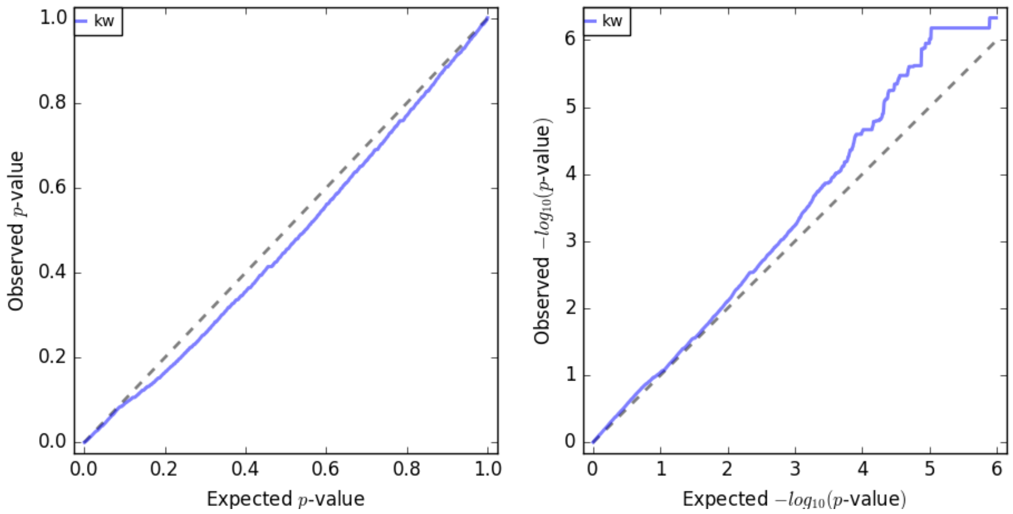

Accelerated Mixed Model

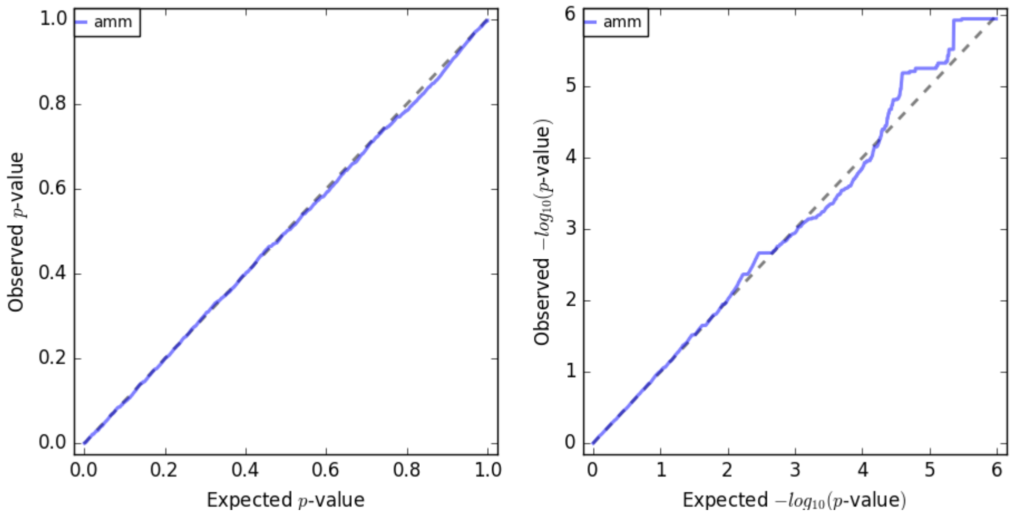

Phase

Linear model

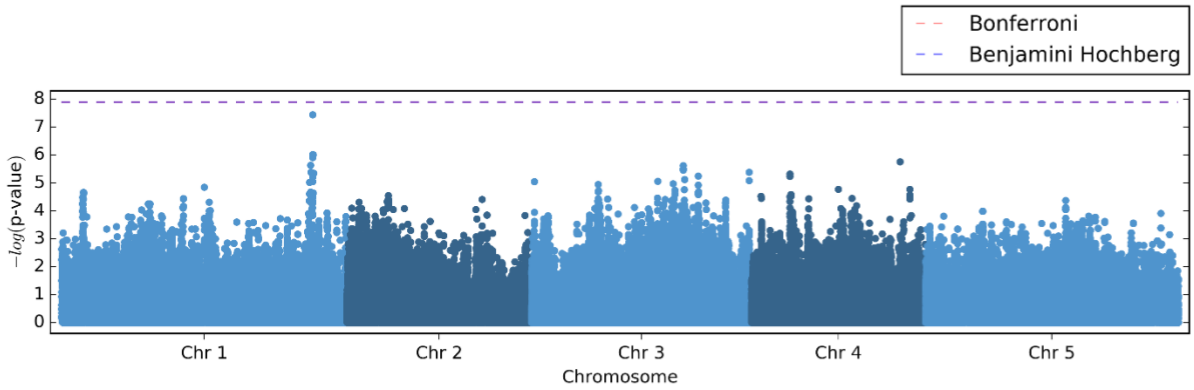

Kruskal-Wallis Model

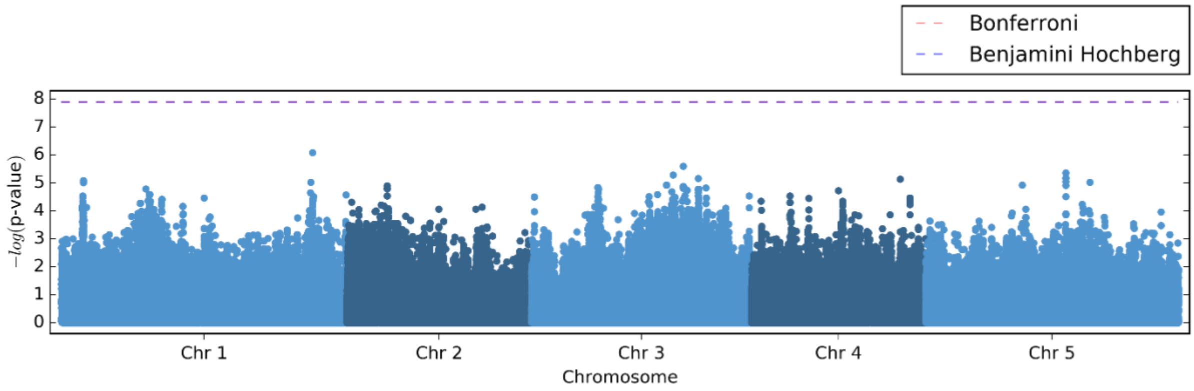

Accelerated Mixed Model

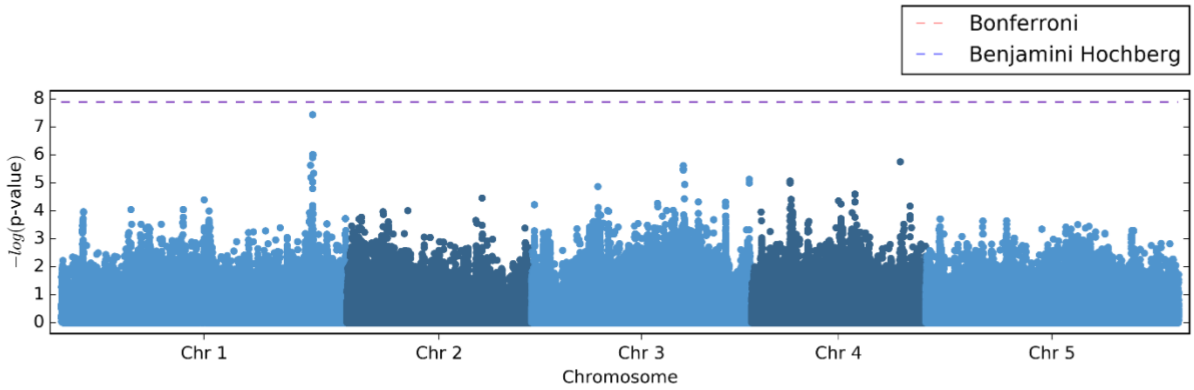

Linear model

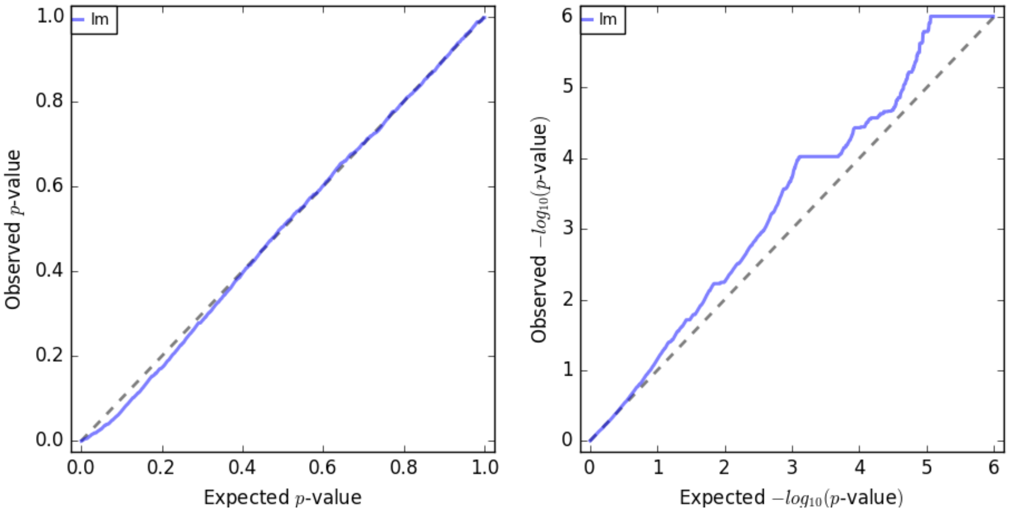

Kruskal-Wallis Model

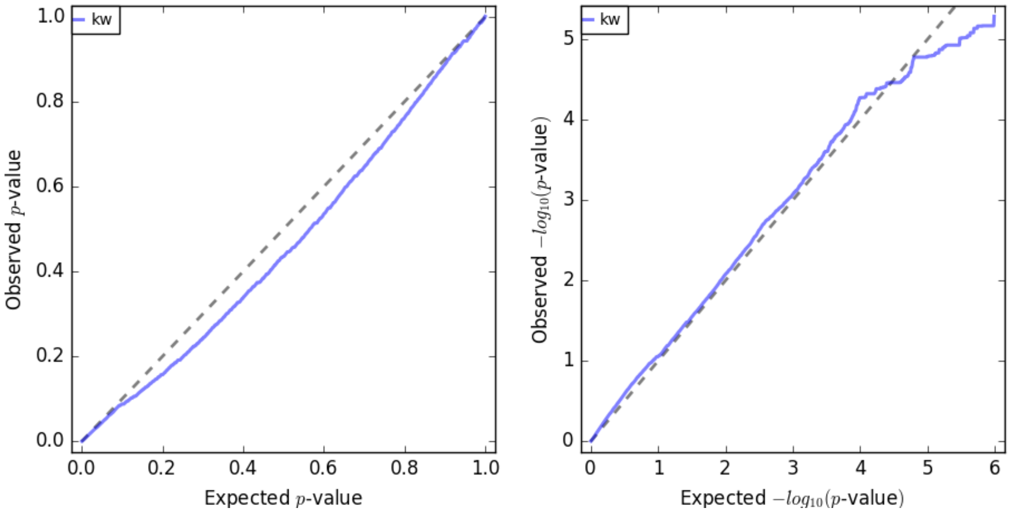

Accelerated Mixed Model

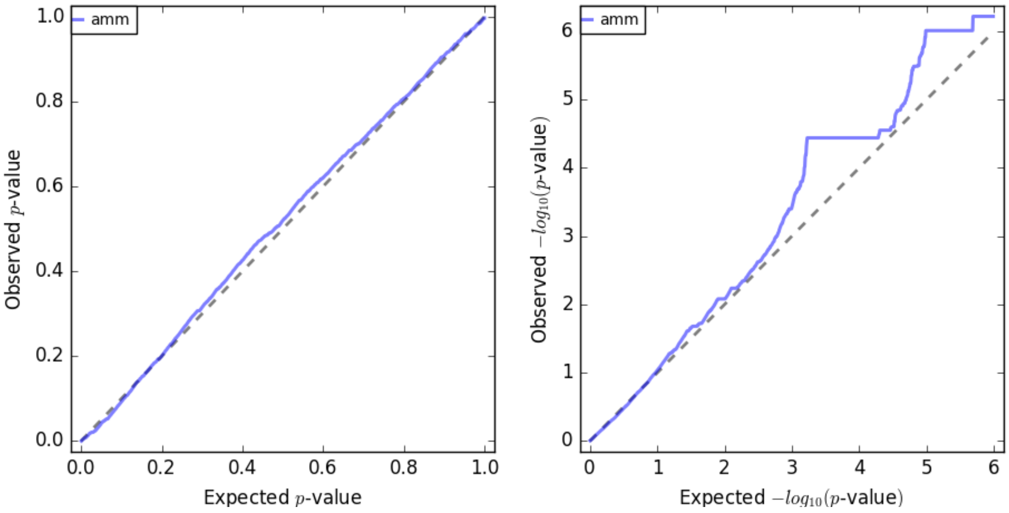

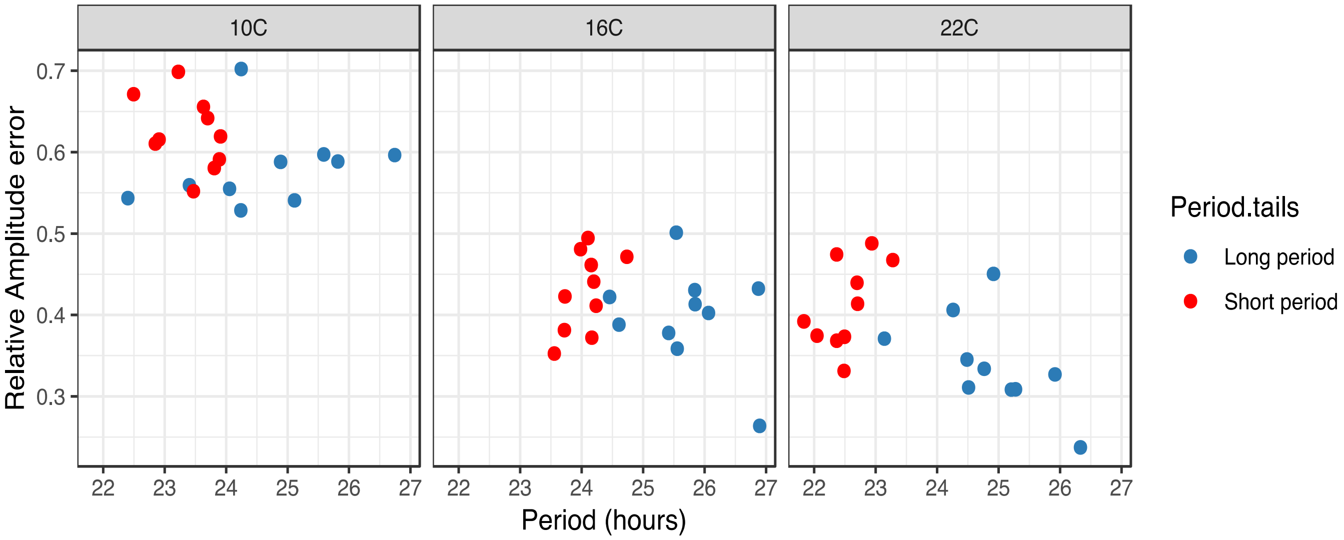

Supplementary Figure 7. Period and RAE correlations in Period tail accessions
Mean periods and RAE values were calculated using a general linear model for each accession in the period tails split across the three temperatures. The two tails remain split in period length across the three temperatures but rhythms become less robust at 10C (higher RAE).

A

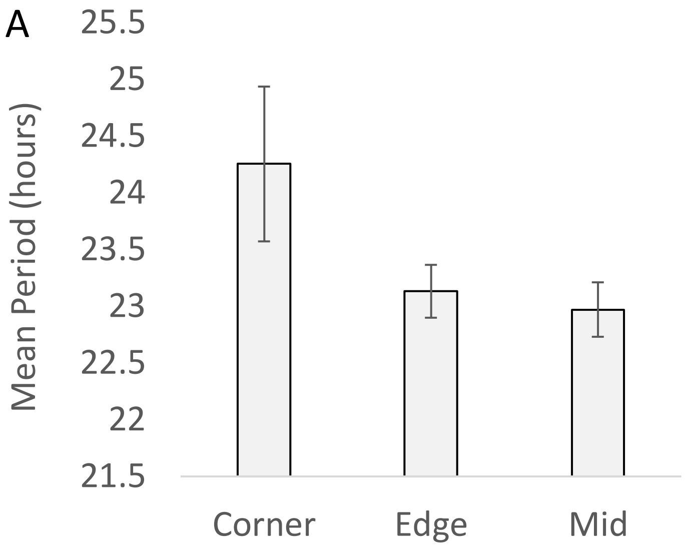

B

	1	2	3	4	5	6	7	8	9	10	11	12
A	23.98	22.95	23.36	22.95	23.67	23.62	23.36	23.29	23.25	22.81	23.42	25.24
B	23.52	23.05	23.37	22.78	22.86	23.26	23.16	22.91	22.61	23.03	22.67	23.26
C	23.28	22.98	23.16	22.73	22.92	22.76	23.29	22.45	23.26	23.12	23.16	23.05
D	22.94	23.06	22.80	22.54	22.86	22.90	22.99	23.00	23.22	22.89	23.58	23.05
E	23.08	23.29	22.85	23.12	22.94	22.72	22.99	22.95	22.94	23.25	22.92	23.13
F	23.10	23.08	22.59	22.68	22.82	23.10	23.15	22.96	23.24	22.97	23.19	23.25
G	22.93	22.83	23.18	22.79	23.16	22.55	22.83	23.53	22.83	22.64	22.85	23.12
H	24.12	22.96	22.81	23.27	22.85	22.96	23.04	22.73	23.08	22.96	23.19	23.68

Supplementary Figure 8. Position in 96-well plate affects period estimation-justification for removing these wells from analysis.
Mean periods for each well of all 96-well plates used in the 191 accession phenotyping dataset were calculated in Genstat. Wells were categorised into ‘corner’ ‘edge’ or ‘middle’ wells and group means were then plotted in the bar chart above (A). Error bars are standard deviation.
Corner wells have much longer period estimates than internal wells irrespective of accession used. Figure B shows all well-means colour-coded for mean period. (Red is long period, purple is short).
Several factors could be affecting period in these wells; they dry out fastest due to exposure to the external atmosphere and less contact with water condensation which could directly influence circadian rhythms. It is also possible that the corner wells are shielded from the overhead light by the side of the plate and so are receiving lower light levels than in the rest of the plate.

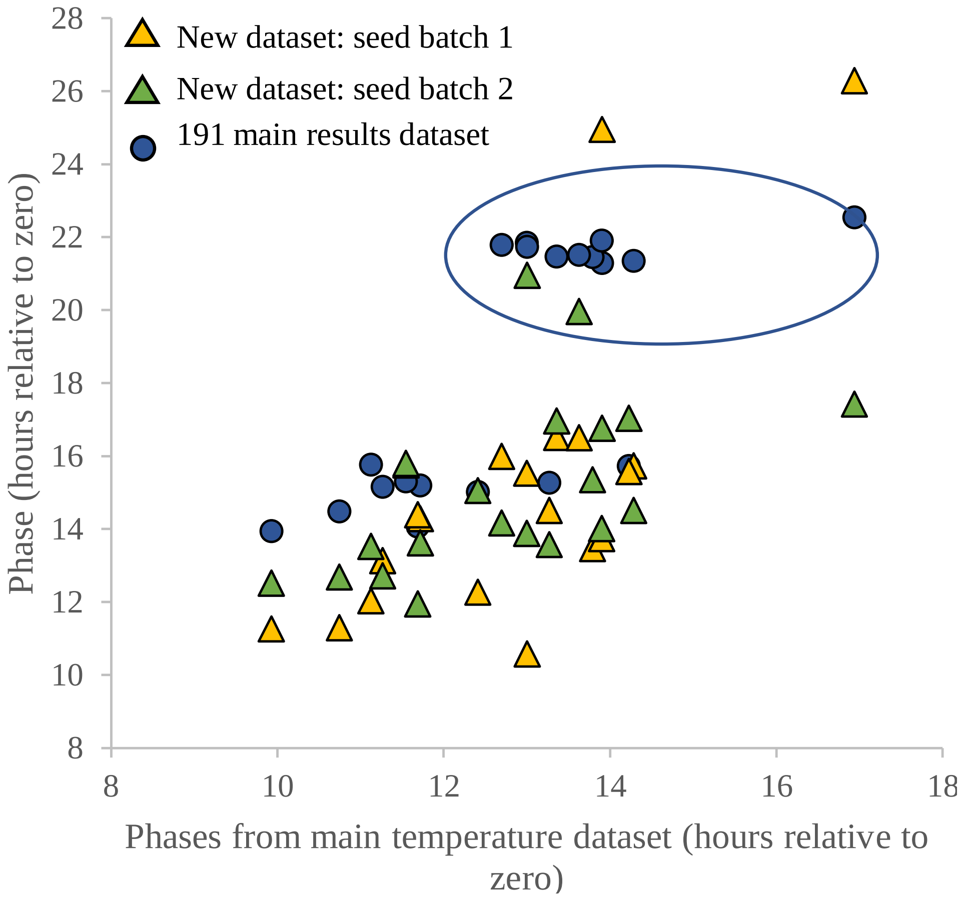

Supplementary Figure 9. Verification of phase predictions at 22C using two seed batches Mean phases for accessions measured at 22°C, against the accession means from the temperature dataset in the main results. Triangle points represent mean phase results from an independent study using seed from the 191 dataset (green triangles) and the temperature dataset (yellow triangles). Means calculated from 7 biological reps per accession per seed batch. Blue circles show accession means from the phase-tail accessions from the 191 dataset in the main text. The 'dawn' accessions from this group (blue oval) have much later phases compared to those estimated from either the temperature dataset in the main results or the new data.

Notes for Supplementary Figure 9: We found that the phase estimates from the phase tail-accessions measured for the 191-accession dataset (Figure 1B, main text) were dawn-shifted compared to those measured in the temperature dataset at 22°C (Figure 5C, main text). We wondered whether this shift was due to using two different seed batches. Using an independent technical replicate, we verified the phases of the accessions in the phase-tail groups. We took seed from the two batches used for the 191 dataset and for the temperature dataset and measured phases under 22°C. There was no significant difference between the phase distributions of the '191-batch' and 'temperature-batch' within the new experiment, (Watson's Two-Sample Test of Homogeneity, t=0.1388, $p > 0.10$). There was a significant difference between the 'dawn' phase tails and the 'dusk' phase tail accessions within the new experiment (Watson's Two-Sample Test of Homogeneity, t=0.6026, $p < 0.001$). This indicates that the differences in phase between the 191 and temperature

datasets is not due to the seed batch used and that there is a difference in phase between the dawn and dusk accessions irrespective of the seed generation.

Supplementary Figure 9 shows the mean phases for accessions measured at 22°C, against the accession means from the temperature dataset presented in Figure 5C of the main text. Phase predictions are generally consistent between experiments, apart from the dawn phased accessions from the 191 dataset. This suggests that when we selected phase tails from the 191 accession data, the accessions we selected as ‘dawn-phased’ had more variable phases, consistent with some of these accessions also having higher RAE means (e.g. Hel-3, St-0 and Lov-1).

REML component effects: Period, RAE, Log10Amplitude

191 Accessions dataset- Y variate: Period						
		Wlad statistic	n.d.f	F statistic	d.d.f	F pr
Fixed effects	Cabinet	22.41	1	22.41	12.2	<0.001
	Run	10.99	2	5.5	12.3	0.02
	Cabinet.Run	9.83	2	4.92	12.2	0.027
Random effects		Variance component	s.e.			
	Replicate	0.022	0.015			
	Replicate.block	0.022	0.015			
	Accession ID	0.611	0.076			
	Sigma squared	1.539	0.046			

Supplementary Table 1. Output from Genstat using the REML directive on 191 period data Using a mixed linear model with cabinet and experimental run as fixed effects and replicate and Accession ID as random effects.

191 Accessions dataset- Y variate: RAE						
		Wlad statistic	n.d.f	F statistic	d.d.f	F pr
Fixed effects	Cabinet	0.07	1	0.07	12.1	0.8
	RUN	11.72	2	5.86	12.1	0.017
	Cabinet.RUN	1.12	2	0.56	12.1	0.585
Random effects		Variance compone nt	s.e.			
	Replicate	0.00018	0.00013			
	Replicate.block	0.00039	0.00015			
	Accession ID	0.00114	0.00022			
	Sigma squared	0.0127	0.00038			

Supplementary Table 2. Output from Genstat using the REML directive on 191 RAE data
Using a mixed linear model with cabinet and experimental run as fixed effects and replicate and Accession ID as random effects.

191 Accessions dataset- Y variate: log10Amplitude						
		Wlad statistic	n.d.f	F statistic	d.d.f	F pr
Fixed effects	Cabinet	2.83	1	2.83	12	0.118
	RUN	377.1	2	188.55	12	<0.001
	Cabinet.RUN	60.58	2	30.29	12	<0.001
Random effects		Variance component	s.e.			
	Replicate	0.0054	0.0038			
	Replicate.block	0.022	0.0038			
	Accession ID	0.0005	0.0012			
	Sigma squared	0.138	0.0042			

Supplementary Table 3. Output from Genstat using the REML directive on 191 log10Amplitude data

Using a mixed linear model with cabinet and experimental run as fixed effects and replicate and Accession ID as random effects.

Likelihood testing: Period, Phase, RAE, Log10Amp

Null Model: Period~Cabinet+Run+Cabinet.Run + Replicate+ Replicate.block
Full Model: Period~Cabinet+Run+Cabinet.Run + Replicate+ Replicate.block + Accession ID

PERIOD							
	d.f.	AIC	Deviance	Chi Sq	logLik	Chi Df	Pr(Chisq)
Null	2516	4489.44	4483.44		-2241.72		
Line added	2515	4035.87	4027.87	455.57	-2013.935	1	<0.0001

Supplementary Table 4. Likelihood test for period
Comparison of two models for period variation with and without Accession ID added as a random effect.

Null Model: Phase~Cabinet+Run
Full Model: Phase~Cabinet+Run+Accession ID

PHASE							
	d.f.	AIC	Deviance	Chi Sq	logLik	Chi Df	Pr(Chisq)
Null	2517	-2419	-2421		1210.5		
Line added	2326	-2824	-2828	407	1414	191	<0.0001

Supplementary Table 5. Likelihood test for phase
Comparison of two models for phase variation with and without Accession ID added as a random effect. Output from LogLikelihood tests in Genstat using Rcircular regression analysis.

Null Model: RAE~Cabinet+Run+Cabinet.Run + Replicate+ Replicate.block
Full Model: RAE~Cabinet+Run+Cabinet.Run + Replicate+ Replicate.block + Accession ID

RAE							
	d.f.	AIC	Deviance	Chi Sq	logLik	Chi Df	Pr(Chisq)
Null	2516	-8156.68	-8162.68		4081.34		
Line added	2515	-8220.65	-8228.65	65.97	4114.325	1	<0.0001

Supplementary Table 6. Likelihood test for RAE
Comparison of two models for RAE variation with and without Accession ID added as a random effect.

Null Model: Log10Amp~Cabinet+Run+Cabinet.Run + Replicate+ Replicate.block
Full Model: Log10Amp ~Cabinet+Run+Cabinet.Run + Replicate+ Replicate.block + Accession ID

LOG10(Amplitude)							
	d.f.	AIC	Deviance	Chi Sq	logLik	Chi Df	Pr(Chisq)
Null	2516	-2219.52	-2225.52		1112.76		
Line added	2515	-2217.71	-2225.71	0.19	1112.855	1	0.663

Supplementary Table 7. Likelihood test for log10Ampltiude
Comparison of two models for log10Ampltiude variation with and without Accession ID added as a random effect.

Supplementary Table 8. Accessions in Period Tails for temperature experiments

Accession ID	Accession name	Short or long tail	Line Mean Period	Line SE Period
8387	St-0	Short	21.278	0.329
6043	Lšv-1	Short	21.459	0.329
6240	TOM 06	Short	21.462	0.300
1552	Sku-30	Short	21.568	0.370
6153	TAA 03	Short	21.612	0.319
6030	Gršn-5	Short	21.651	0.309
6201	TDr-16	Short	21.723	0.341
9343	Dju-1	Short	21.725	0.292
9433	Nyl 13	Short	21.762	0.387
6238	TOM 04	Short	21.837	0.341
9404	HolA-1 1	Long	24.229	0.309
9453	Stenk-2	Long	24.267	0.309
6022	Fjä2-6	Long	24.331	0.329
6413	Ull3-4	Long	24.357	0.292
6096	T1060	Long	24.388	0.319
6035	Hov1-10	Long	24.448	0.319
6114	T570	Long	24.678	0.285
6149	T970	Long	24.955	0.387
6123	T680	Long	25.539	0.319
6133	T800	Long	25.700	0.355

Supplementary Table 9. Accessions in Phase Tails for temperature experiments

Accession ID	Accession name	Dusk or Dawn phase Tail	Line Mean circular Phase	Line SE Phase
6011	Eden-6	Dusk	12.21	0.58
6123	T680	Dusk	13.93	0.23
6133	T800	Dusk	14.07	0.23
6124	T690	Dusk	14.48	0.21
6038	Hov3-5	Dusk	15.01	0.24
9381	Fri 1	Dusk	15.16	0.20
6035	Hov1-10	Dusk	15.19	0.21
9332	Bar 1	Dusk	15.27	0.21
6132	T790	Dusk	15.30	0.27
6149	T970	Dusk	15.72	0.22
6043	Löv-1	Dawn	21.28	0.20
6013	Eden-9	Dawn	21.34	0.29
6069	Nyl-7	Dawn	21.45	0.19
8230	Algutsrum	Dawn	21.47	0.21
6074	Ör-1	Dawn	21.51	0.20
9433	Nyl 13	Dawn	21.74	0.23
1552	Sku-30	Dawn	21.78	0.21
6036	Hov3-2	Dawn	21.85	0.21
8387	St-0	Dawn	21.90	0.20
9402	Hel-3	Dawn	22.54	0.20

Supplementary Table 10. Accessions in RAE Tails for temperature experiments

Accession ID	Accession name	RAE: low and high	Line Mean RAE	Line SE RAE
6123	T680	Low	0.310999	0.023396
6114	T570	Low	0.316548	0.021708
1063	Brösarp-21-140	Low	0.326776	0.021708
5832	App1-16	Low	0.338294	0.021707
9404	HolA-1 1	Low	0.343043	0.022936
6126	T720	Low	0.34418	0.023396
6218	TFÄ 08	Low	0.344506	0.021708
6173	TÅD 05	Low	0.344854	0.022936
9383	Fri 3	Low	0.346338	0.021708
6095	T1050	Low	0.347766	0.022503
6210	TEDEN 03	High	0.426781	0.025563
6238	TOM 04	High	0.428421	0.024406
6010	Eden-5	High	0.42886	0.02621
8387	St-0	High	0.430485	0.023885
6043	Löv-1	High	0.438462	0.023884
9436	Puk-1	High	0.438879	0.022935
992	Ale-Stenar-44-4	High	0.439084	0.025561
6070	Omn-1	High	0.441196	0.026206
6209	TEDEN 02	High	0.441694	0.024406
9402	Hel-3	High	0.443792	0.023883

Trait		Freezing tolerance		Flowering time at 10C		Flowering time at 16C		Flowering time (Mean in Swedish environment)		Flowering time (Mean in Spanish environment)		Seed dormancy	
Source		Horton 2016		Sasaki 2015		Sasaki 2015		Li 2010		Li 2010		Kerdaffrec 2016	
Number common accessions		107		127		127		29		29		123	
Circadian traits		R2	P	R2	P	R2	P	R2	P	R2	P	R2	P
Period		0.008	0.352	0.027	0.063	0.001	0.708	0.227	**0.009**	0.163	0.030	0.008	0.352
Phase		0.010	0.317	0.045	0.017	0.009	0.299	0.256	**0.005**	0.241	**0.007**	0.010	0.317
RAE		0.006	0.446	0.005	0.417	0.000	0.838	0.058	0.208	0.020	0.461	0.006	0.446

Supplementary Table 11. Linear regression with previously published datasets. Significant *p*-values (<0.01) are highlighted in red. Data from Sasaki et al was measured under long days (16h-8h) and constant temperatures in controlled growth cabinets. Data from Li et al is the mean of four conditions replicating either Swedish or Spanish weather patterns across two years.

Mutant validation tables

Supplementary Table 12: Testing differences in Period (Welch Two Sample t-test)

Plant ID	Sample size across all reps	Period mean (hours)	Period SD	t value	df	p value
**col-0**	180	**24**	**2.02**			
cor27-1	108	24	1.75	0.18	118	0.857
cor27-2	107	24.2	2.21	-0.35	142	0.726
cor28-2	158	25.3	1.78	-3.86	117	>0.001
cor28-2/27-1	101	26.5	2.33	-6.24	139	>0.001
mybl2	103	24	1.97	-0.06	140	0.955
parc6	97	23.9	2.08	0.36	138	0.722
parc6-1	104	23.4	1.77	1.93	120	0.056
atg1g71015	102	23.7	2.11	0.96	138	0.338
**WT for cor28-1**	72	**23.9**	**1.62**			
cor28-1	70	25.3	1.73	2.41	39	0.021
**ler**	108	**23.2**	**2.55**			
sco2	101	23.7	1.95	-0.61	92	0.546

Supplementary Table 13: Testing differences in RAE (Welch Two Sample t-test)

Plant ID	N	RAE mean	RAE SD	t value	df	p-value
**col-0**	180	**0.42**	**0.18**			
cor27-1	108	0.34	0.18	3.7612	132.65	>0.001
cor27-2	107	0.29	0.14	6.1329	109.28	>0.001
cor28-2	158	0.28	0.13	6.2523	104.52	>0.001
cor28-2/27-1	101	0.28	0.14	5.7682	120.7	>0.001
mybl2	103	0.37	0.17	2.6062	128.76	0.01024
parc6	97	0.37	0.16	3.0367	126.56	0.00291
parc6-1	104	0.35	0.17	3.6523	130.41	>0.001
atg1g71015	102	0.38	0.16	2.6593	124.82	>0.001
**WT for cor28-1**	72	**0.33**	**0.17**			
cor28-1	70	0.28	0.16	-0.87301	36.889	0.3883
**ler**	108	**0.43**	**0.17**			
sco2	101	0.51	0.16	-1.7234	120.37	0.08738

Supplementary Table 14: Testing differences in Phase (Watson's Two-Sample Test of Homogeneity)

Plant ID	N	Circular Phase mean (hours)	Phase SD	test statistic	P value
**col-0**	180	15.35	1.04		

cor27-1	108	13.39	0.77	0.4726	< 0.001
cor27-2	107	14.02	0.83	0.2467	< 0.05
cor28-2	158	12.93	0.91	0.3669	< 0.01
cor28-2/27-1	101	12.67	0.98	0.6332	< 0.001
mybl2	103	14.27	0.93	0.0911	> 0.10
parc6	97	13.91	0.97	0.0886	> 0.10
parc6-1	104	14.95	0.87	0.0599	> 0.10
atg1g71015	102	14.45	0.92	0.1455	> 0.10
**WT for cor28-1**	72	14.54	0.79		
cor28-1	70	12.95	0.86	0.1941	< 0.05
**ler**	108	16.37	1.04		
sco2	101	15.33	1	0.0568	> 0.10

Supplementary Table 15. Accumulated analysis of varience table for period with temperature data

Period						
**(Cabinet+Temp)*Period_tails/real_name**						
Accumulated analysis of variance						
Change	degrees of freedom	Sum of squares	Mean Square	Variance ratio	F probablility	Inference
Cabinet	1	52.621	52.621	10.71	0.001	There is some difference in period between cabinets but the Cabinet.Temp combinations are not replicated so cannot control as random effect
Temp	2	331.625	165.812	33.74	<.001	Very significant effect of temperature on period
Period_tails	1	1008.214	1008.214	205.15	<.001	Very significant effect of tails group on period
Cabinet*Period_tails	1	49.504	49.504	10.07	0.002	Some evidence that tails behave differently in the two cabinets
Temp*Period_tails	2	70.998	35.499	7.22	<.001	Very significant; behaviour of tails differs across the three temperatures
Period_tails*real_name	18	414.268	23.015	4.68	<.001	Significant difference between period of accessions within each tail group
Cabinet*Period_tails*real_name	18	70.574	3.921	0.80	0.705	The difference between accessions within each tail group does not vary between cabinets
Temp*Period_tails*real_name	36	241.081	6.697	1.36	0.076	The difference between accessions within each tail group does not vary between temperatures
Residual	1146	5632.036	4.915			
Total	1225	7870.920	6.425			
NOTES:Period difference due to the Period_tail groups is greater than the effects due to Temperature. The two tails behave differently in response to temperature						

Supplementary Table 16. Accumulated analysis of varience table for RAE with temperature data

**RAE**						
**(Cabinet+Temp)*RAE_tails/real_name**						
Accumulated analysis of variance						
Change	degrees of freedom	Sum of squares	Mean Square	Variance ratio	F probablility	Inference
Cabinet	1	0.2799	0.2799	9.52	0.002	There is some difference in rae between cabinets but the Cabinet.Temp combinations are not replicated so cannot control as random effect
Temp	2	11.20351	5.60176	190.48	<.001	Very significant effect of temperature on RAE
RAE_tails	1	2.08938	2.08938	71.05	<.001	Very significant effect of tails group on RAE
Cabinet*RAE_tails	1	0.08555	0.08555	2.91	0.088	Tails behave similarly within the two cabinets
Temp*RAE_tails	2	0.68898	0.34449	11.71	<.001	Tails behave differently at the three temperatures
RAE_tails*real_name	18	2.21867	0.12326	4.19	<.001	Significant difference between RAE of accessions within each tail group
Cabinet*RAE_tails*real_name	18	0.40046	0.02225	0.76	0.753	The difference between accessions within each tail group does not vary between cabinets
Temp*RAE_tails*real_name	36	1.29203	0.03589	1.22	0.176	The difference between accessions within each tail group does not vary between temperatures
Residual	1148	33.76123	0.02941			
Total	1227	52.0197	0.0424			
NOTES:Temperature affects RAE more than the tail groups						

Supplementary Table 17. Circular regression analysis for Phase with temperature data

**Phase**							
**Circular regression analysis**							
Response variate: Degrees							
Distribution: von Mises							
Link function: Mu = Mu0 + 2*ARCTAN(lp)							
Number of units: 1107							
**Fitted terms: Cabinet + Temp + Phase_tails + Temp.Phase_tails +Phase_tails.real_name + Temp.Phase_tails.real_name**							
Phase circular regression	removed term	d.f	Devience	Diffierence in Devience	difference in d.f	P-value	Inference
Full model:Cabinet + Temp + Phase_tails + Temp.Phase_tails +Phase_tails.real_name + Temp.Phase_tails.real_name		55	-790.4				
Cabinet + Temp + Phase_tails + Temp.Phase_tails +Phase_tails.real_name	Temp.Phase_tails.real_name	23	-725.2	65.2	32	<0.001	Significant variation in response of phase to temperature within the two Phase_tail groups
Cabinet + Temp + Phase_tails + Temp.Phase_tails	Phase_tails.real_name	7	-691	34.2	16	<0.01	Some diff between lines within each Phase tail group
Cabinet + Temp + Phase_tails	Temp.Phase_tails	5	-689.7	1.3	2	0.52	No sig diff in responses of the Phase_tail groups to Temp on average (even though lines within tail groups vary).
Cabinet + Temp	Phase_tails	4	-656.4	33.3	1	<0.00001	Hugely sig diff on average between Phase_tails
Temp	Cabinet	3	-653.7	2.7	1	0.1	Weak evidence for any effect of Cabinet
Null	Temp			231.7338	3	<0.00001	This deviance gives you the effect of Temp (the only thing left in the model). Hugely significant
NOTES: Although there is a large difference between the two phase groups, they do not behave diffierently to temperature							
