# Supplementary figures and images for: Naturally occurring circadian rhythm variation associated with clock gene loci in Swedish Arabidopsis accessions
Source: Plant Cell Environ. 2021 Jan 11;44(3):807–20. doi: 10.1111/pce.13941 (PMC7986795; doi:10.1111/pce.13941)

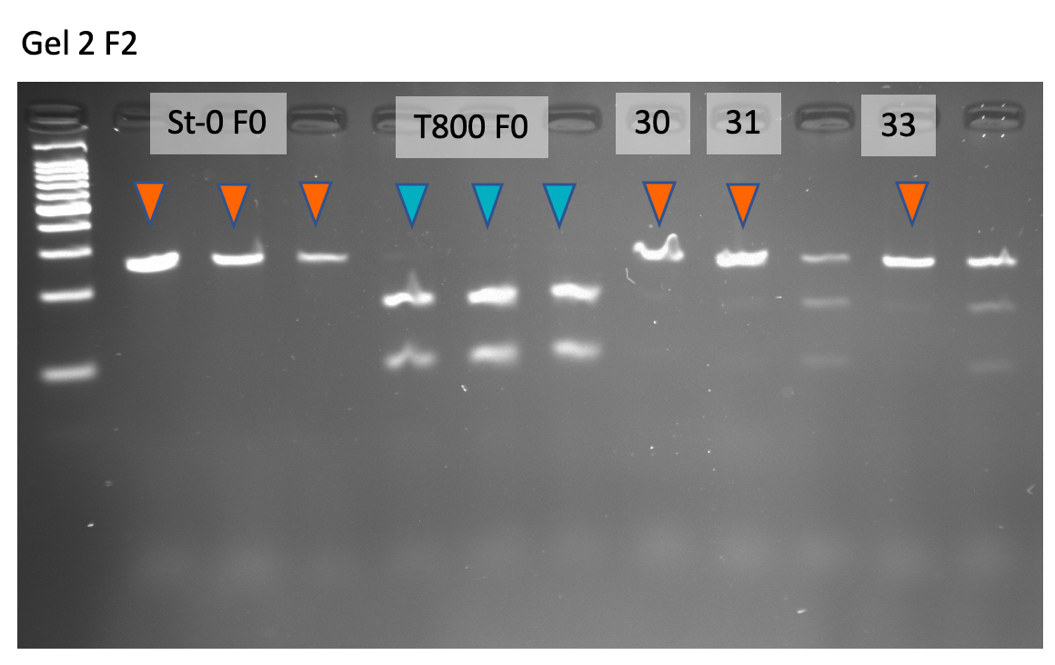

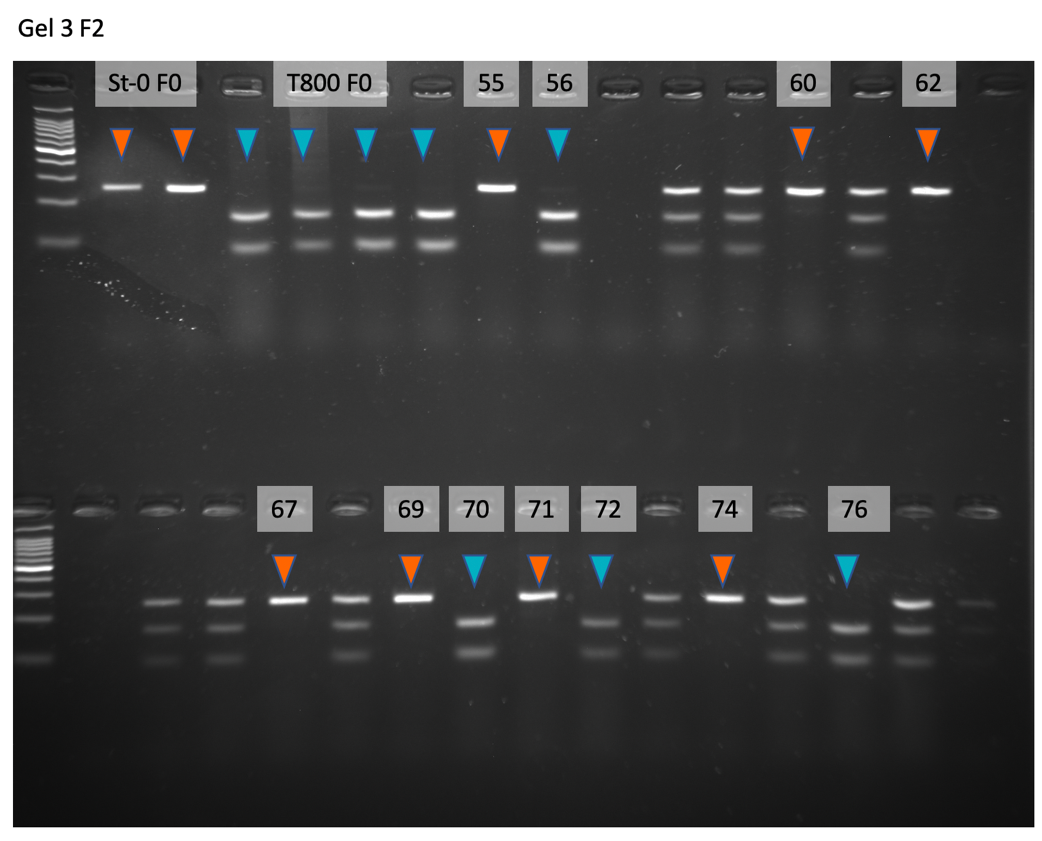

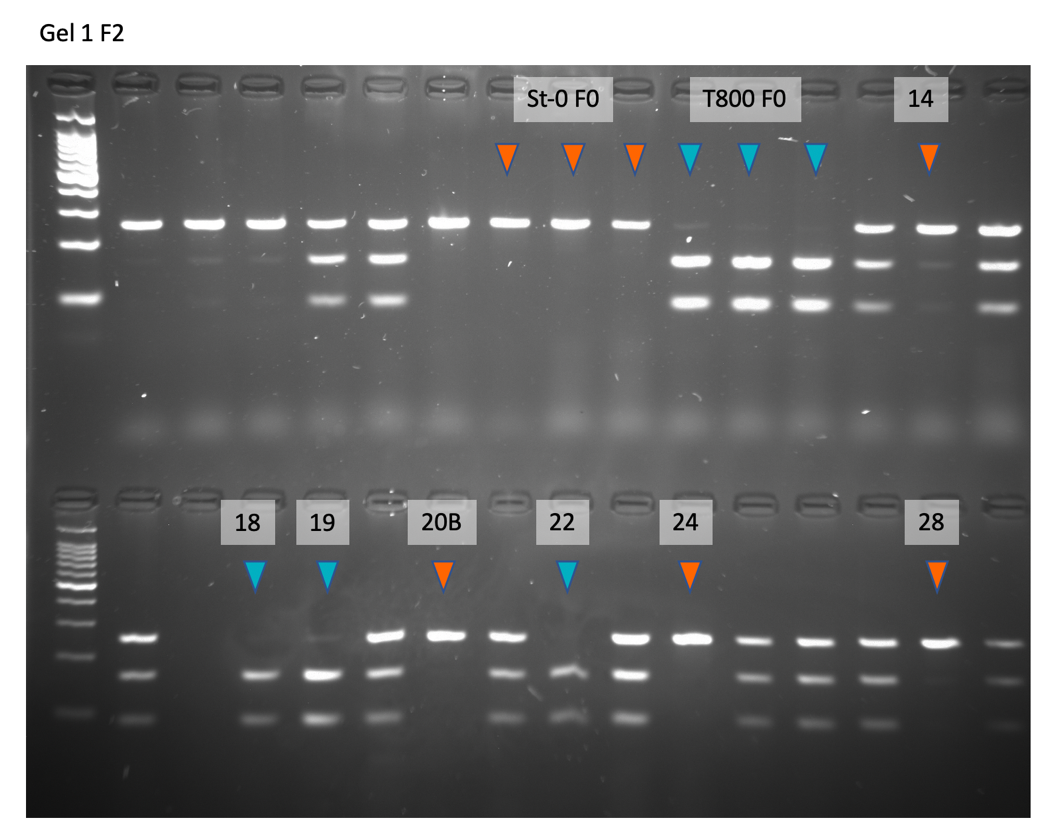


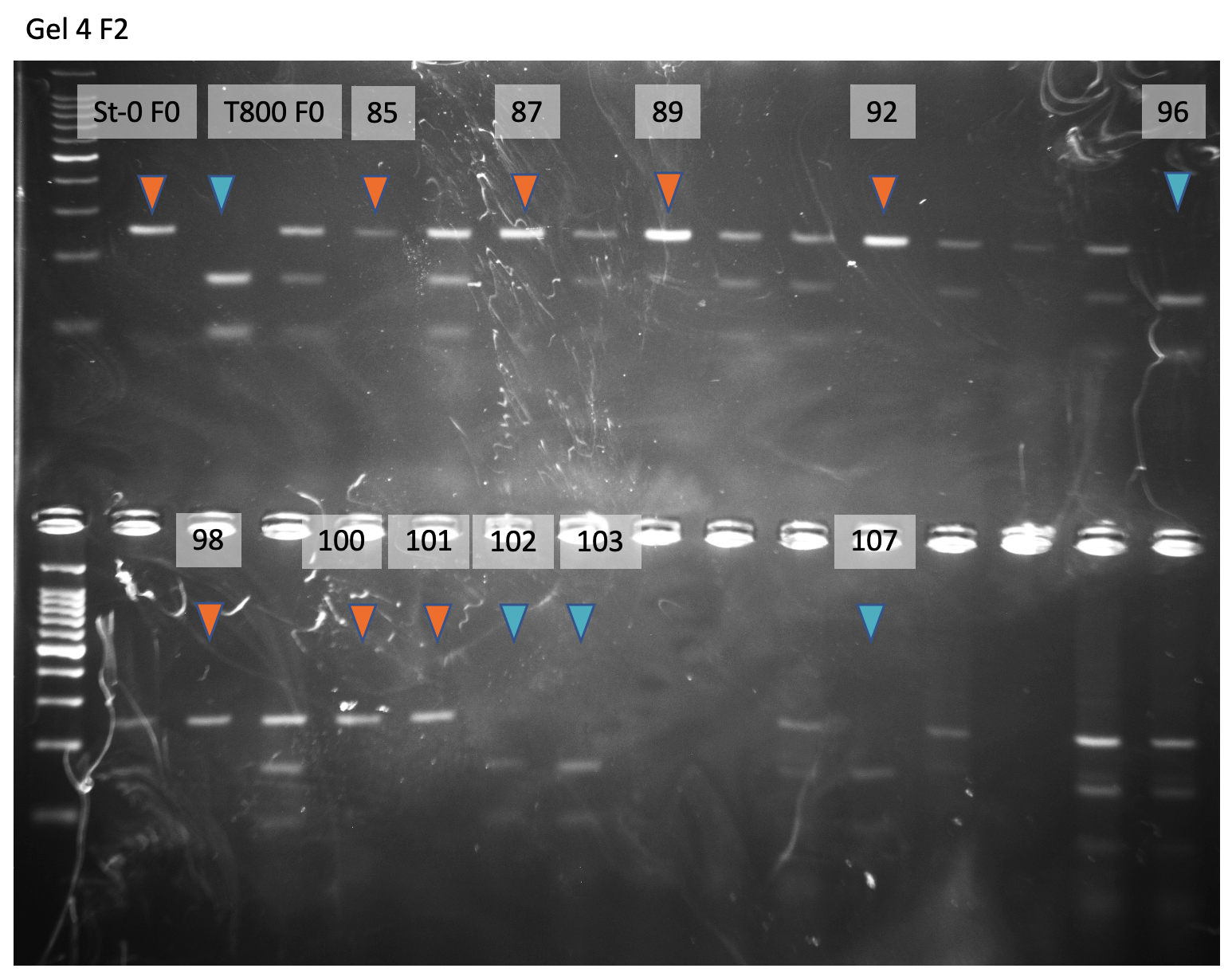

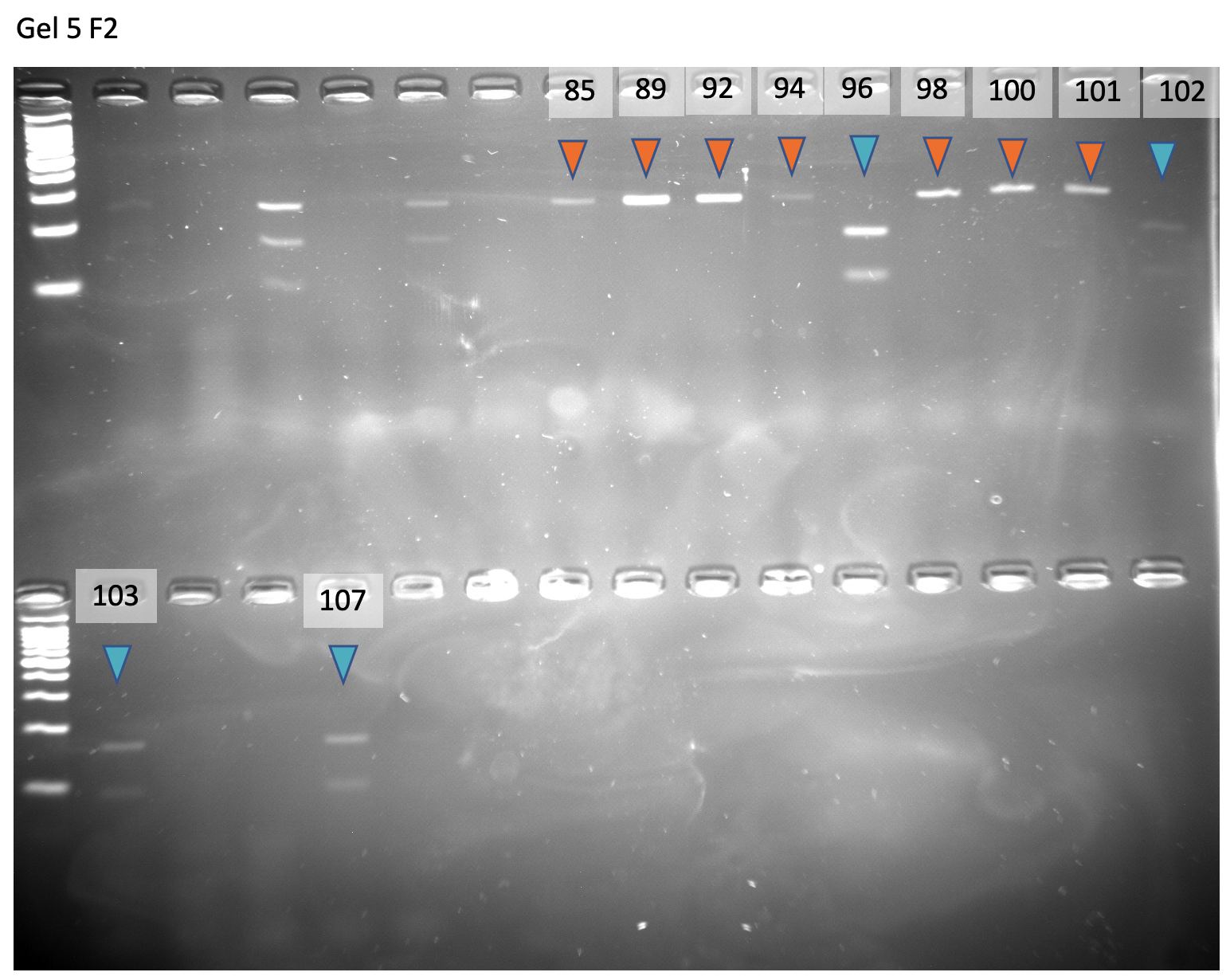

Supplement: Supplementary file 8 — Supplementary File 6 Gels from CAPS assay for co‐segregation analysis [file PCE-44-807-s006.docx]
